# New 3-Ethynylaryl Coumarin-Based Dyes for DSSC Applications: Synthesis, Spectroscopic Properties, and Theoretical Calculations

**DOI:** 10.3390/molecules26102934

**Published:** 2021-05-14

**Authors:** João Sarrato, Ana Lucia Pinto, Gabriela Malta, Eva G. Röck, João Pina, João Carlos Lima, A. Jorge Parola, Paula S. Branco

**Affiliations:** 1 LAQV-REQUIMTE, Departament of Chemistry, NOVA School of Science and Technology, FCT NOVA, Universidade NOVA de Lisboa, 2829-516 Caparica, Portugal; j.sarrato@campus.fct.unl.pt (J.S.); al.pinto@campus.fct.unl.pt (A.L.P.); g.malta@campus.fct.unl.pt (G.M.); lima@fct.unl.pt (J.C.L.); 2 Department of Chemistry, Coimbra Chemistry Centre, University of Coimbra, Rua Larga, 3004-535 Coimbra, Portugal; eva.roeck@dcb.unibe.ch (E.G.R.); jpina@qui.uc.pt (J.P.); 3 Department of Chemistry, Biochemistry and Pharmaceutical Sciences, University of Bern, Freiestrasse 3, CH-3012 Bern, Switzerland

**Keywords:** dye-sensitized solar cells, coumarin dyes, thieno [3,2-*b*] thiophene, charge transfer, ethynylaryl

## Abstract

A set of 3-ethynylaryl coumarin dyes with mono, bithiophenes and the fused variant, thieno [3,2-*b*] thiophene, as well as an alkylated benzotriazole unit were prepared and tested for dye-sensitized solar cells (DSSCs). For comparison purposes, the variation of the substitution pattern at the coumarin unit was analyzed with the natural product 6,7-dihydroxycoumarin (Esculetin) as well as 5,7-dihydroxycomarin in the case of the bithiophene dye. Crucial steps for extension of the conjugated system involved Sonogashira reaction yielding highly fluorescent molecules. Spectroscopic characterization showed that the extension of conjugation via the alkynyl bridge resulted in a strong red-shift of absorption and emission spectra (in solution) of approximately 73–79 nm and 52–89 nm, respectively, relative to 6,7-dimethoxy-4-methylcoumarin (λ_abs_ = 341 nm and λ_em_ = 410 nm). Theoretical density functional theory (DFT) calculations show that the Lowest Unoccupied Molecular Orbital (LUMO) is mostly centered in the cyanoacrylic anchor unit, corroborating the high intramolecular charge transfer (ICT) character of the electronic transition. Photovoltaic performance evaluation reveals that the thieno [3,2-*b*] thiophene unit present in dye **8** leads to the best sensitizer of the set, with a conversion efficiency (η = 2.00%), best V_OC_ (367 mV) and second best J_sc_ (9.28 mA·cm^−2^), surpassed only by dye **9b** (J_sc_ = 10.19 mA·cm^−2^). This high photocurrent value can be attributed to increased donor ability of the 5,7-dimethoxy unit when compared to the 6,7 equivalent (**9b**).

## 1. Introduction

Following O’Regan and Grätzel’s seminal application [1] of an organic dye adsorbed on a mesoporous wide band gap semiconductor as a light-harvesting electrode, the field of DSSCs (Dye-Sensitized Solar Cells) has garnered much interest over the last three decades. Compared to alternative light-harvesting technologies, they possess reduced cost, ease of manufacture and low environmental impact, which combined with their wide array of possible colors and compatibility with flexible substrates, allows for a multitude of applications, such as integration into buildings [2,3] and interiors [4,5].

Although the original ruthenium(II)-polypyridyl chromophores used in DSSCs, such as N3 [6] and N719 [7], have mostly remained as gold standard dyes due to their large conversion efficiencies [8], their only moderate extinction coefficients and the use of a rare and expensive noble metal has led to the extensive search for highly efficient metal-free dyes. These metal-free dyes offer, in addition to the lower cost of production, easier and greater synthetic versatility and tunable optical and electrochemical properties through structural modification, as demonstrated by the extremely wide array of compounds explored over the years [9,10,11]. Despite being mostly overshadowed by their inorganic counterparts, in recent years comparable or even superior performances have been achieved by metal-free chromophores, such as bulky indoline-quinoxaline dyes (10.65%) [12], tetrathioacene dyes (10.1%) [13] and more elaborate polycyclic aromatic push-pull dyes (12.6%) [14], all employing a Co(II/III) complex as redox shuttle. Additionally, the use of co-sensitization approaches has been employed to great success with organic dyes, managing to reach efficiencies of more than 14% [15,16].

To be adequately applied in DSSCs, organic dyes must present a donor-π-acceptor (D-π-A) structure. The push-pull effect in these D-π-A dyes leads to efficient intramolecular charge transfer (ICT) from the donor to the acceptor unit through the π-bridge upon light absorption. Among the many classes of organic compounds used, coumarins are of particular interest due to their wide use as fluorescent sensors [17,18,19], emitting layers in Organic Light-Emitting Diodes (OLEDs) [20,21,22] and in laser applications [23,24], owing to their large Stokes shift, high quantum yields and good solubility. Additionally, their photophysical properties can be easily tuned through the addition of substituents, namely electron-withdrawing substituents in position 3 and electron-donating substituents in position 7 [25].

This allows for a decrease in the energy gap between the highest occupied molecular orbital (HOMO) and the lowest unoccupied molecular orbital (LUMO), making coumarins great candidates as new sensitizers for DSSCs. Hara et al. [26,27,28] were some of the first to successfully design and employ dyes with coumarin donor units to achieve competitive efficiencies of up to 8.2% [29] when using deoxycholic acid (DCA) as a coadsorbent. In more recent work, Jiang et al. [30] and He et al. [31] used additional indoline and triphenylamine donors (respectively) attached to the coumarin unit, while Vekariya [32] investigated the effect of various *o*-halide phenylene spacers on dye structure and device performance.

Recently we have shown that the introduction of a linear ethynyl π-bridge into coumarin-based conjugated donor–acceptor systems resulted in redshifted absorption and emission spectra relatively to the styryl counterparts [33]. Electrochemical studies revealed that this derivatization resulted in a marked decrease in the HOMO energy levels, which influenced the overall conversion efficiency with a significantly superior performance. This revealed the importance of the alignment of the substituents with the direction of the intramolecular charge transfer. This is not completely surprising since the incident photon-to-current conversion efficiency (IPCE) reveals that the electron transfer yield (Φ(ν)ET) becomes larger with the introduction of a triple bond [34].

**Figure 1:**
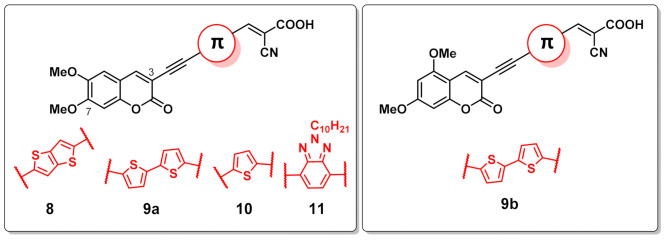
Structure of target dyes prepared and characterized in this work.

Following up on our previous experience with the synthesis of coumarin chromophores [33,35,36], several 3-ethynyl-6,7-dihydroxycoumarin-based dyes were prepared, with emphasis on the effect of varying the π-bridge on dye structure, photophysical properties and sensitizer efficiency. These groups include mono and bithiophenes, including a fused variant, thieno [3,2-*b*] thiophene, as well as a benzotriazole unit containing a long alkyl chain (Figure 1). Additionally, a 5,7-dihydroxycoumarin-based chromophore was prepared, to ascertain the effect of this alternative substitution pattern on device performance. All prepared dyes were then spectroscopically characterized, their optimized geometry was obtained from density functional theory (DFT) and their performance as sensitizers was evaluated.

## 2. Results and Discussion

### 2.1. Synthesis and Characterization

**Scheme 1:**
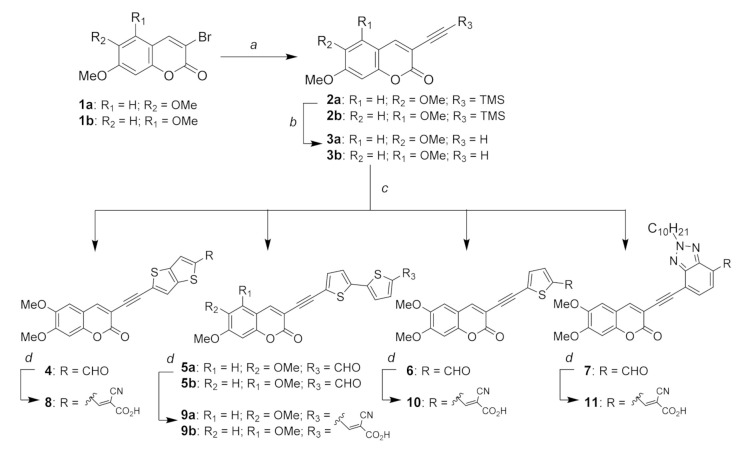
Synthetic approach used for the preparation of the various chromophores: (**a**) **1a**/**1b** (1 eq.) ethynyltrimethylsilane (2 eq.), Pd(PPh_3_)_4_ (0.15 eq.), PPh_3_ (0.06 eq.), CuI (0.12 eq.), (*i*-Pr) _2_NH (2 eq.), dry dioxane, sealed tube under N_2_, 40–45 °C, overnight; (**b**) **2a**/**2b** (1 eq.), K_2_CO_3_ (0.15 eq.), dry MeOH, r.t., 4h; (**c**) ethynylcoumarin (1 eq.), aldehyde (1 eq.), Pd(PPh_3_)_4_ (0.15 eq.), PPh_3_ (0.06 eq.), CuI (0.12 eq.), (*i*-Pr) _2_NH (2 eq.), dry dioxane, sealed tube under N_2_, 40–45 °C, overnight; (**d**) aldehyde (1 eq.), cyanoacetic acid (3 eq.), piperidine (2.7 eq.), dry acetonitrile, reflux, overnight.

The synthetic approach to obtain the coumarin-based dyes, as described in Scheme 1, initiated with the brominated derivatives **1a** and **1b**. Compound **1a** was obtained as described by Martins et al. [36], while **1b** was prepared through condensation of ethyl propiolate and 1,3,5-trihydroxybenzene [37], followed by chloroformylation, bromination [38], hydrolysis and methylation. The 3-ethynyl coumarin derivatives (**3a** and **3b**) were then obtained through a high-yield Sonogashira coupling with ethynyltrimethylsilane, followed by the removal of the trimethylsilyl group, which at first was accomplished with tetrabutylammonium fluoride (TBAF). Since this method resulted in poor yields, partly due to the possible degradation of the unprotected ethynyl derivative, a method employing K_2_CO_3_ in MeOH described by Wang et al. [39] was used instead, without further purification of the resulting product.

The heterocyclic π-bridges, in the form of brominated aldehydes, were then coupled to the ethynyl moiety through another Sonogashira coupling. In the case of compounds **4** and **7** the aldehydes were not commercially available and as such were prepared by lithiation-formylation of 2,5-dibromothieno [3,2-*b*] thiophene in the case of compound **4**, and alkylation, double bromination [40] and lithiation-formylation of benzotriazole in the case of compound **7**.

With the aldehyde groups now present in the molecules, a Knoevanagel condensation is performed as described by Martins et al. [33] in order to insert the cyanoacrylic acid group that allows the binding to the TiO_2_ surface. With this, the final cromophores **8**, **9a**, **9b**, **10** and **11** were obtained with overall yields of 20.2%, 20.3%, 1.1%, 23.6% and 3.6%, respectively. The coumarin derivative with substitution at the 5 and 7-positions (as in compound **9b**) proved to be more difficult to handle then their 6,7-disubstituted analogues, due to insolubility problems. This drawback, together with the increased synthetic complexity of the 5,7-dihydroxycoumarin dye system, led us to focus on the 5,7-disubstitued derivatives.

### 2.2. Absorption and Fluorescence

**Figure 2:**
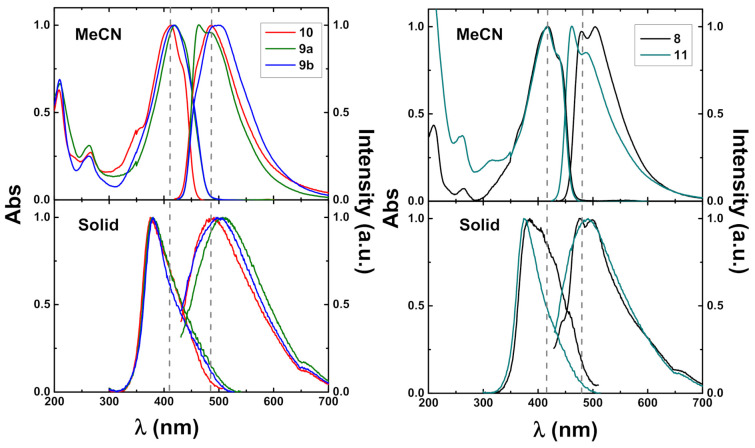
Normalized absorption and fluorescence emission spectra for compounds **8**–**11** in acetonitrile solution and in the solid state (adsorbed in TiO_2_ films) at 293K.

Figure 2 presents the UV-Vis absorption and fluorescence emission spectra of the investigated samples at room temperature in acetonitrile solution. The significant Stokes shift values (in the 2201–3998 cm^−1^ range) point to a charge transfer (CT) character of the fluorescence emission band (Table 1). Indeed, when compared to 6,7-dimethoxy-4-methylcoumarin (λ_abs_ = 341 nm, λ_em_ = 410 nm in ethanol) the absorption and emission spectra of samples **8**–**11** are strongly redshifted [41]. This is associated with the increase in conjugation length of the chromophoric system due to overlapping of π-orbitals of the coumarin group with the π-orbitals of the cyanoacetic acid substituted benzotriazole or thienyl units. Moreover, the absorption spectra of samples **8**–**11** are also redshifted when comparison is made with 7-methoxy-3-acetylcoumarin (λ_abs_ = 341 nm) where intramolecular charge transfer (ICT) was found due to the introduction of the acetyl group in the 3-position of the coumarin moiety [42]. This behavior is explained by the increasing ICT character of compounds **8**–**11**, promoted by the strong electron-withdrawing character of the cyanoacetic acid substituted benzotriazole or thienyl units, thus leading to high ground-state dipole moments (values in the 14.560–24.230 D range, see Table 2).

Porous TiO_2_ films (about 1 μm thick) were made by spreading TiO_2_ paste (ref. 30NR-D, from GreatcellSolar) onto electrically conductive Fluorine-Doped Tin Oxide (FTO) glass, using Scotch Magic tape as a spacer. The film paste was then gradually sintered up to 500 °C to reach the anatase TiO_2_ phase. When the films had cooled to about 80 °C, they were placed for 1 min in concentrated solutions of the samples **8**–**11** in acetonitrile solution (0.1 mM) for adsorption. The absorption (here measured by the fluorescence excitation spectra) and fluorescence emission spectra are depicted in Figure 2. In general, the spectra of the investigated samples in the solid state are blue-shifted by 31–41 nm, with respect to the absorption spectra in MeCN solutions.

### 2.3. Theoretical Calculations

The ground state optimized geometry structures and the relevant HOMO and LUMO energy levels, together with their electron density distribution surface plots were obtained at the DFT/CAM-B3LYP/6-311G(d,p) level taking into account the bulk solvent effects of acetonitrile. Frequency analysis for each compound were also computed and did not yield any imaginary frequencies, indicating that the structure of each molecule corresponds to at least a local minimum on the potential energy surface. The geometry optimization for the investigated compound revealed that in general the thienyl and/or the benzotriazole moieties are mostly planar with the coumarin units.

The optimized ground-state molecular geometries found for the investigated compounds were used to obtain the vertical excitation energies, oscillator strengths (f) and excited state compositions in terms of excitations between the occupied and virtual orbitals using the time-dependent density functional theory (TD-DFT) approach, see Table 2. For samples **8**–**11** the predicted S_0_→S_1_ transitions are in good agreement with the observed lowest energy absorption bands in acetonitrile solution (Table 1). In general, for these transitions the major contribution arises from the HOMO→LUMO orbitals (contributions > 78%). It is worth mentioning that the calculated absorption band gap values agree with the experimental values (see Table 2), thus giving support for the predicted ground-state geometries.

**Figure 3:**
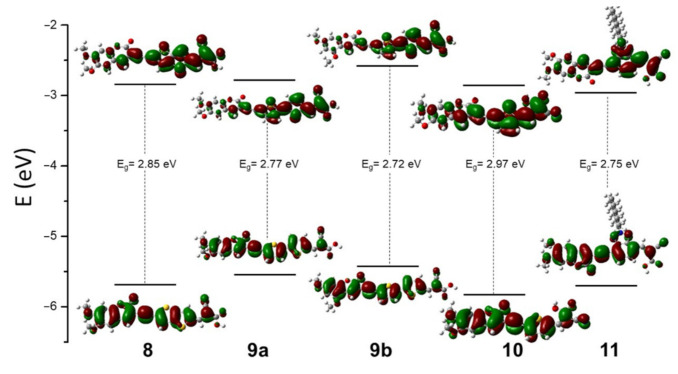
DFT//CAM-B3LYP/6311G(d,p) optimized ground-state geometry together with the frontier molecular orbital energy levels and the relevant electronic density contours (calculated at B3LYP/6311G(d,p) level) for the investigated compounds. Additionally, displayed are the predicted optical band gap energy values, E_g_.

The molecular orbital contours (Figure 3) show that the densities of the HOMO orbitals are, in general, spread over the entire molecules, while the LUMO shows a decrease in the electron density on the coumarin moieties and a concomitant increase in the benzotriazole or thienyl units. The electronic delocalization in the LUMO orbitals gives support for the occurrence of a charge transfer state (CT) in the singlet excited state.

### 2.4. Electrochemical Characterization

The electrochemical properties of the dyes were determined by differential pulse voltammetry (DPV), with the main results being presented in Table 3 (See Supplementary Materials for full DPV data). From the obtained onsets of oxidation and reduction peaks, the HOMO and LUMO energies were estimated. For this purpose, the following equation was used: E [eV] = −(E_onset_ (V vs. SCE) + 4.44) [43].

All calculated band gap values are similar across the various dyes, arising from similar values in the HOMO (−5.74 to −5.60 eV) and in the LUMO energies (−3.62 to −3.53eV) energies. These differences are within the error for electrochemical determination of the energy of frontier orbitals (~0.1 V) [43], and as such a comparison between the dyes cannot be confidently made. The determined electrochemical band gaps in DMF (~2.1 eV) are lower than the corresponding optical band gaps (~2.7 eV, Table 2), determined in acetonitrile. Solvation and coulombic effects are responsible for often observed differences between electrochemical and optical band gaps [44]. Additionally, the solvatochromic nature of substituted coumarins [45,46] leads to a shorter band gap in more polar solvent, which is the case with DMF.

When compared with other reported coumarin sensitizers (E_HOMO_: −5.2 eV; E_LUMO_: −2.4 eV) [30], a significant decrease in orbital energy is observed for the dihydroxycoumarin dyes. This is indicative of a lower electron-donating ability of the dihydroxy substitution pattern in comparison with the indoline moieties employed in the referenced work. On the other hand, this family of dyes presents a shorter band gap (2.0 V vs. 2.4 V), a direct consequence of the more extensive conjugation of the π-system.

### 2.5. Photovoltaic Performance

**Figure 4:**
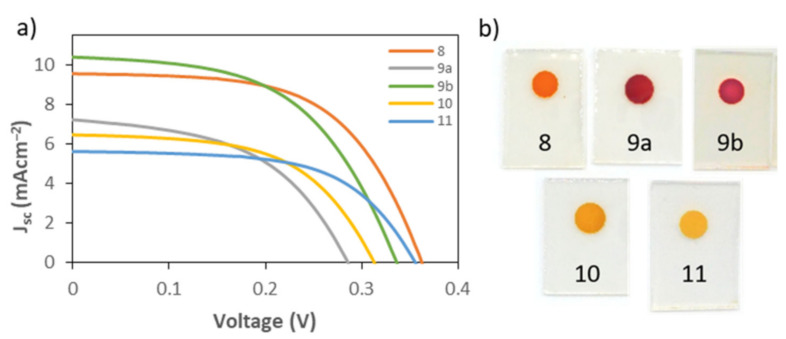
(**a**) I–V curves of the test cells based on the synthesized dyes under 100 mW·cm^−2^ under simulated AM 1.5 illumination. The results presented correspond to the best performing cell. (**b**) Pictures of the dyes adsorbed on the TiO_2_ photoanodes.

The prepared chromophores were tested in prototype devices and their performance was compared with reference dye N719 (in non-optimized conditions). These results are summarized in Figure 4 and Table 4. 

Compound **8** was the best performing dye, with an efficiency of 2% and the highest values of V_OC_ and V_max_, 367 and 256 mV, respectively. This marked difference from the other dyes can be attributed to the comparably higher ε value (6.4470 cm^−1^M^−1^), as well as the more redshifted absorption when adsorbed on the TiO_2_ surface (386 nm). Dye **9a**, which contains a 2,2′-bithiophene group as π-bridge, obtained the worst efficiency value (0.95%), comparable to dye **10** (1.07%) containing only one thiophene ring. One possible explanation for this result may be the known torsion angles present between the two thiophene units [47], which are not present in the fused ring equivalent **8**, and result in hindered conjugation. This possibility seems to be further supported by the Stokes shift observed for this dye (∆_SS_ = 2201 cm^−1^), being the lowest of the group, which indicates a low ICT character for electronic transition.

Comparison of dyes **9a** and **9b** allows the evaluation of the effect of the position of the substituents on DSSC performance, and it is immediately apparent from the obtained efficiencies (0.95% vs. 1.78%, respectively) that position 5 (when compared to 6) is a superior choice for donor units in coumarin dyes. The superior efficiency is a consequence of the high photocurrent values (J_sc_ = 10.2 and J_max_ = 7.8 mA·cm^−2^), which even surpass the results for dye **8**, allowing it to have a good efficiency despite the relatively modest photovoltage (V_oc_ = 339 and V_max_ = 227 mV). The improved properties of position 5 as a donor are further supported by the obtained absorption properties, in particular the absorptivity (ε = 4.5480 cm^−1^M^−1^) and Stokes shift (∆_SS_ = 3998 cm^−1^), which are indicative of the dye’s effective light absorption and charge transfer character. Additionally, dye **9b** possesses a higher HOMO energy than dye **9a**, which once again points to position 5 of the coumarin unit being a more suitable choice for the inclusion of electron-donating groups.

Inversely, dye **11** shows a high V_OC_, comparable to dye **8** (359 vs. 367 mV, respectively), yet it has the worst J_sc_ (5.4 mA·cm^−2^) of all the synthesized dyes, which can be due to its inferior ε (12,060 cm^−1^M^−1^) and Stokes shift (∆_SS_ = 2451 cm^−1^). The photovoltage values can be attributed to the bulky alkyl chain present in the benzotriazole moiety, which will help suppress charge recombination between the semiconductor and the oxidized redox shuttle [48,49]. Another effect of the presence of this bulky group may be the distortion of the molecule’s geometry [50], decreasing planarity and therefore leading to a less efficient electron delocalization and lower ICT character in the transition. Once again, this is reflected in the observed Stokes shift value (∆_SS_ = 2451 cm^−1^), which is on the lower end of the group, comparable to dye **9a**.

## 3. Materials and Methods

### 3.1. General Information and Instruments

All solvents and reagents were obtained commercially (Merck KGaA, Darmstadt, Germany) and used without further purification. The drying of the solvents was achieved with M2A molecular sieves (Merck KGaA), as described by Bradley et al. [51].

Thin-layer chromatography (TLC) was carried out on aluminum-backed Kieselgel 60 F254 silica gel plates (Merck KGaA). Plates were visualized with UV light (254 and 336 nm) and in certain cases chemical staining agents (Acidic solution of 2,4-dinitrophenylhidrazine). Preparative-layer chromatography (PLC) was performed on Keiselgel 60 F254 silica gel plates (Merck KGaA) with a thickness of 0.5 mm. Column chromatography was performed using Keiselgel 60 silica gel (Merck KGaA), 70–230 mesh and 230–400 mesh particle sizes as stationary phases, in the cases of regular and flash [52] normal-phase cromatographies, respectively.

The ^1^H- and ^13^C-NMR (nuclear magnetic spectroscopy) spectra were acquired with a Bruker Avance III 400 (Billerica, MA, USA), at 400 and 101 MHz, respectively. Absorption and fluorescence spectra were recorded on a Cary 5000 UV-Vis-NIR (Santa Clara, CA, USA) and Horiba–Jobin–Ivon Fluoromax4 spectrometers (Longjumeau, France), respectively. The fluorescence spectra were corrected for the wavelength response of the system. 

Differential pulse voltammetry (DPV) measurements were performed on a μAutolab Type III potentiostat/galvanostat (Metrohm Autolab B. V., Utrecht, The Netherlands), controlled with GPES (General Purpose Electrochemical System) software version 4.9 (Eco-Chemie, B. V. Software, Utrecht, The Netherlands), using a cylindrical 5 mL three-electrode cell. All measurements refer to a saturated calomel electrode (SCE, saturated KCl) reference electrode (Metrohm, Utrecht, The Netherlands). A Pt wire was used as counter-electrode, a glassy carbon electrode (MF-2013, f = 1.6 mm, BAS inc., West Lafayette, IN, USA) was used as the working electrode. Prior to use, the working electrode was polished in aqueous suspensions of 1.0 and 0.3 mm alumina (Buehler, Esslingen, Germany) over 2–7/′′ micro-cloth (Buehler) polishing pads, then rinsed with water and ethanol. This cleaning procedure was systematically applied before any electrochemical measurement. The electrolyte composition was 0.1 M tetrabutylammonium tetrafluoroborate in DMF, with a dye concentration of 1.5 × 10^−4^ M. Measurements were performed between 0 and +1.6 V for determination of oxidation potential and between 0 and −1.6 V for determination of reduction potential, with a scan rate of 10 mV/s in both cases. The samples in the electrochemical cell were de-aerated by purging with nitrogen for 10 min prior to, and during, the electrochemical measurements.

High-resolution mass spectra (HRMS) were obtained at the University of Porto, Mass Spectrometry Laboratory (LEM/CEMUP) using a mass spectrometer Linear Trap Quadrupole (LTQ) Orbitrap XL^TM^ (Thermo Fischer Scientific, Bremen, Germany) controlled by a LTQ Tune Plus 2.5.5 and Xcalibur 2.1.0 and at the University of Salamanca (Spain), Elemental Analysis, Chromatography and Mass Spectrometry Service (NUCLEUS), using a High Performance Liquid Chromatography (HPLC) Agilent 1100 coupled to a QSTAR XL Hybrid qTOF (AB Sciex, Framingham, MA, USA) mass spectrometer.

### 3.2. Synthesis

#### 3.2.1. Synthesis of 6,7-Dimethoxy-3-((Trimethylsilyl)Ethynyl)Coumarin (**2a**) and 5,7-Dimethoxy-3-((Trimethylsilyl)Ethynyl)Coumarin (**2b**)

To a sealed tube, 0.06 eq. of PPh_3_, 0.12 eq. of CuI, 0.15 eq. of Pd(PPh_3_)_4_, 1 eq. of 3-bromocoumarin (**2a**/**2b**) and 5 mL of dry dioxane were added under a N_2_ atmosphere. After a few minutes, 2 eq. of ethynyltrimethylsilane and 0.35 mL 2 eq. of dry (*i*-Pr)_2_NH were added and the solution was stirred at 45 °C overnight under a N_2_ atmosphere. Once the reaction was confirmed to be complete by TLC (hexane/AcOEt (7:3 *v*/*v*)), the solution was cooled to room temperature, the solvent was removed under reduced pressure and the solid residue dried in vacuo before being purified by flash chromatography with hexane/AcOEt (7:3 *v*/*v*) as eluent, affording the target compounds.

*6,7-dimethoxy-3-((trimethylsilyl)ethynyl)coumarin* (**2a**). Starting from 347.3 mg (1.22 mmol, 1 eq.) of 3-bromo-6,7-dimethoxycoumarin (**1a**), 343.4 mg (93.2%) of 6,7-dimethoxy-3-((trimethylsilyl)ethynyl)coumarin (**2a**) were obtained. ^1^H-NMR (400 MHz, CDCl_3_) δ (ppm) 7.83 (s, 1H, H4), 6.82 (s, 1H, H5/H8), 6.80 (s, 1H, H5/H8), 3.95 (s, 3H, H1′/H2′), 3.91 (s, 3H, H1′/H2′), 0.26 (s, 9H, H5′); ^13^C-NMR (101 MHz, CDCl_3_) δ (ppm) 160.0 (C2), 153.5 (C7), 149.8 (C8a), 146.8 (C4/C6), 146.1 (C4/C6), 111.5 (C3/C4a/C5), 109.5 (C3/C4a/C5), 107.7 (C3/C4a/C5), 101.0 (C3′/C4′/C8), 99.9 (C3′/C4′/C8), 98.7 (C3′/C4′/C8), 56.6 (C1′/C2′), 56.5 (C1′/C2′), −0.1 (C5′); HRMS-ESI(+) Calculated for C_16_H_19_O_4_Si [M + H]^+^ 303.1047; Found 303.1053.

*5,7-dimethoxy-3-((trimethylsilyl)ethynyl)coumarin* (**2b**). Starting from 147.1 mg (1.22 mmol, 1 eq.) of 3-bromo-5,7-dimethoxycoumarin (**1b**), 114.0 mg (72.9%) of 5,7-dimethoxy-3-((trimethylsilyl)ethynyl)coumarin (**2b**) were obtained. ^1^H-NMR (400 MHz, CDCl_3_) δ (ppm) 8.14 (s, 1H, H4), 6.37 (s, 1H, H6/H8), 6.25 (d, *J* = 2.4 Hz, 1H, H6/H8), 3.88 (s, 3H, H1′/H2′), 3.84 (s, 3H, H1′/H2′), 0.25 (s, 9H, H5′). HRMS-ESI(+) Calculated for C_16_H_19_O_4_Si [M + H]^+^ 303.1047; Found 303.1041.

#### 3.2.2. General Method for the Synthesis of Coupled Aldehydes (**4**–**7**)

To a sealed tube, PPh_3_ (0.06 eq), CuI (0.12 eq), Pd(PPh_3_)_4_ (0.15), aldehyde (1 eq.) and 5 mL of dry dioxane were added under a N_2_ atmosphere. After a few minutes ethynylcoumarin (**3**) (1 eq.) and dry (*i*-Pr)_2_NH (2 eq.) were added and the solution was stirred at 45°C overnight under a N_2_ atmosphere. Once the reaction was confirmed to be complete by TLC (hexane/AcOEt (7:3 *v*/*v*)), the solution was cooled to room temperature, the solvent was removed under reduced pressure and the solid residue dried in vacuo before being purified by flash chromatography with DCM/MeOH (99.8:0.02 *v*/*v* and 99.5:0.05 *v*/*v*) as eluent, affording a bright yellow solid in all cases.

*5-((6,7-dimethoxy-2-oxo-2H-chromen-3-yl)ethynyl)thieno [3,2-b] tiophene-2-carbaldehyde* (**4**). Starting from 104.6 mg (0.42 mmol, 1 eq.) of 5-bromothieno [3,2-*b*] thiophene-2-carbaldehyde, 89.5 mg (51.5%) of 5-((6,7-dimethoxy-2-oxo-2*H*-chromen-3-yl)ethynyl)thieno [3,2-*b*] tiophene-2-carbaldehyde (**4**) were obtained. ^1^H-NMR (400 MHz, CDCl_3_) δ (ppm) 9.99 (s, 1H, H7′), 7.92 (s, 1H, H4), 7.89 (s, 1H, H5′/H6′), 7.54 (s, 1H, H5′/H6′), 6.87 (s, 1H, H5/H8), 6.86 (s, 1H, H5/H8), 3.98 (s, 3H, H1′/H2′), 3.94 (s, 3H, H1′/H2′); HRMS-ESI(+) Calculated for C_20_H_13_O_5_S_2_ [M + H]^+^ 397.0199; Found 397.0197.

*5′-((6,7-dimethoxi-2-oxo-2H-chromen-3-yl)ethynyl)-[2,2′-bithiophene]-5-carbaldehyde* (**5a**). Starting from 132.8 mg (0.49 mmol, 1 eq.) of 5-bromo-[2,2′-bithiophene]-5-carbaldehyde, 95.2 mg (46.3%) of 5′-((6,7-dimethoxi-2-oxo-2*H*-chromen-3-yl)ethynyl)-[2,2′-bithiophene]-5-carbaldehyde (**5a**) were obtained. ^1^H-NMR (400 MHz, CD_2_Cl_2_) δ (ppm) 9.85 (s, 1H, H9′), 7.89 (s, 1H, H4), 7.71 (d, *J* = 4.8 Hz, 1H, H5′/H6′/H7′/H8′), 7.30 (s, 3H, H5′/H6′/H7′/H8′), 6.87 (s, 1H, H5/H8), 6.85 (s, 1H, H5/H8), 3.92 (s, 3H, H1′/H2′), 3.87 (s, 3H, H1′/H2′); HRMS-ESI(+) Calculated for C_22_H_15_O_5_S_2_ [M + H]^+^ 423.0355; Found 423.0348.

*5′-((5,7-dimethoxi-2-oxo-2H-chromen-3-yl)ethynyl)-[2,2′-bithiophene]-5-carbaldehyde* (**5b**). Starting from 154.0 mg (0.56 mmol, 1 eq.) of 5-bromo-[2,2′-bithiophene]-5-carbaldehyde, 22.4 mg (14.1%) of 5′-((5,7-dimethoxi-2-oxo-*2H*-chromen-3-yl)ethynyl)-[2,2′-bithiophene]-5-carbaldehyde (**5b**) were obtained. ^1^H-NMR (400 MHz, DMF-d_7_) δ (ppm) 10.02 (s, 1H, H9′), 8.29 (s, 1H, H4), 8.08 (d, *J* = 4.03 Hz, 1H, H5′/H6′/H7′/H8′), 7.69–7.67 (m, 2H, H5′/H6′/H7′/H8′), 7.53 (d, *J* = 4.03 Hz 1H, H5′/H6′/H7′/H8′), 6.67 (s, 1H, H6/H8), 6.63 (s, 1H, H6/H8), 4.04 (s, 3H, H1′/H2′), 3.99 (s, 3H, H1′/H2′); HRMS-ESI(+) Calculated for C_22_H_15_O_5_S_2_ [M + H]^+^ 423.0355; Found 423.0349.

*5-((6,7-dimethoxy-2-oxo-2H-chromen-3-yl)ethynyl)-thiophene-2-carbaldehyde* (**6**). Starting from 64.8 mg (0.34 mmol, 1 eq.) of 5-bromothiophene-2-carbaldehyde, 58.7 mg (50.8%) of 5-((6,7-dimethoxy-2-oxo-2*H*-chromen-3-yl)ethynyl)-thiophene-2-carbaldehyde (**6**) were obtained. ^1^H-NMR (400 MHz, CD_2_Cl_2_) δ (ppm) 9.87 (s, 1H, H7′), 7.95 (s, 1H, H4), 7.71 (d, *J* = 3.8 Hz, 1H, H5′/H6′), 7.41 (d, *J* = 3.7 Hz, 1H, H5′/H6′), 6.89 (s, 1H, H5/H8), 6.87 (s, 1H, H5/H8), 3.94 (s, 3H, H1′/H2′), 3.89 (s, 3H, H1′/H2′); HRMS-ESI(+) Calculated for C_18_H_13_O_5_S [M + H]^+^ 341.0478; Found 341.0478.

*2-decyl-7-((6,7-dimethoxy-2-oxo-2H-chromen-3-yl)ethynyl)-2H-benzo[d][1,2,3]triazole-4-carbaldehyde* (**7**). Starting from 113 mg (0.31 mmol, 1 eq.) of 7-dibromo-2-decyl-2*H*-benzo[*d*][1,2,3]triazole-4-carbaldehyde, 42.5 mg (50.8%) of 2-decyl-7-((6,7-dimethoxy-2-oxo-2*H*-chromen-3-yl)ethynyl)-2*H*-benzo[*d*][1,2,3]triazole-4-carbaldehyde (**7**) were obtained. 1H-NMR (400 MHz, CD2Cl2) δ (ppm) 10.50 (s, 1H, H7′), 8.11 (s, 1H, H4), 7.99 (d, *J* = 7.3 Hz, 1H, H5′/H6′), 7.81 (d, *J* = 7.5 Hz, 1H, H5′/H6′), 6.96 (s, 1H, H5/H8), 6.93 (s, 1H, H5/H8), 4.90 (t, *J* = 7.4 Hz, 2H, H1′′), 3.99 (s, 3H, H1′/H2′), 3.94 (s, 3H, H1′/H2′), 2.26–2.18 (m, 2H, H2′′), 1.43–1.30 (m, 15H, H3′′-H9′′), 0.90 (t, *J* = 6.4 Hz, 3H, H10′′); HRMS-ESI(+) Calculated for C_30_H_34_N_3_O_5_ [M + H]^+^ 516.2493; Found 516.2509.

#### 3.2.3. General Method for the Synthesis of Final Chromophores (**8**–**11**)

To a round-bottom flask containing aldehyde (1 eq.), cyanoacetic acid (3 eq.), 5 mL of acetonitrile (ACN) and dry piperidine (2.7 eq.) were added and the resulting solution was stirred under reflux for 24 h. Once the reaction was confirmed to be complete by TLC (DCM/MeOH (9.5:0.5 *v*/*v*)), the solvent was evaporated under reduced pressure, the solid residue was washed 3–5 times with ACN, acidified with HCl (10%) and washed 3–5 times with distilled water. After each washing step the solvent used was centrifuged (4500 rpm, 10–30 min) to recover any lost product.

*2-cyano-3-(5-((6,7-dimethoxy-2-oxo-2H-chromen-3-yl)ethynyl)thieno[3,2-b]thiophen-2-yl)acrylic acid* (**8**). Starting from 15 mg (0.038 mmol, 1 eq.) of 5-((6,7-dimethoxy-2-oxo-2*H*-chromen-3-yl)ethynyl)thieno[3,2-*b*]tiophene-2-carbaldehyde (**4**), 7.8 mg (44.5%) of 2-cyano-3-(5-((6,7-dimethoxy-2-oxo-2*H*-chromen-3-yl)ethynyl)thieno[3,2-*b*]thiophen-2-yl)acrylic acid (**8**) were obtained. ^1^H-NMR (400 MHz, DMSO-*d*_6_) δ (ppm) 8.75 (br s, 1H, OH), 8.37 (s, 1H, H4/H7′), 8.28 (s, 1H, H4/H7′), 8.09 (s, 1H, H5′/H6′), 7.89 (s, 1H, H5′/H6′), 7.26 (s, 1H, H5/H8), 7.14 (s, 1H, H5/H8), 3.90 (s, 3H, H1′/H2′), 3.82 (s, 3H, H1′/H2′). HRMS-ESI(+) Calculated for C_23_H_14_NO_6_S_2_ [M + H]^+^ 464.0257; Found 464.0249.

*2-cyano-3-(5′-((6,7-dimethoxy-2-oxo-2H-chromen-3-yl)ethynyl)-[2,2′-bithiophen]-5-yl)acrylic acid* (**9a**). Starting from 95 mg (0.225 mmol, 1 eq.) of 5′-((6,7-dimethoxi-2-oxo-2*H*-chromen-3-yl)ethynyl)-[2,2′-bithiophene]-5-carbaldehyde (**5a**), 54.7 mg (49.7%) of 2-cyano-3-(5′-((6,7-dimethoxy-2-oxo-2*H*-chromen-3-yl)ethynyl)-[2,2′-bithiophen]-5-yl)acrylic acid (**9a**) were obtained. ^1^H-NMR (400 MHz, DMF-*d*_7_) δ (ppm) 8.57 (s, 1H, H4/H9′), 8.38 (s, 1H, H4/H9′), 8.08 (d, *J* = 3.5 Hz, 1H, H5′/H6′/H7′/H8′), 7.73 (d, *J* = 3.7 Hz, 1H, H5′/H6′/H7′/H8′), 7.70 (d, *J* = 4.0 Hz, 1H, H5′/H6′/H7′/H8′), 7.53 (d, *J* = 4.5 Hz, 1H, H5′/H6′/H7′/H8′), 7.37 (s, 1H, H5/H8), 7.15 (s, 1H, H5/H8), 4.02 (s, 3H, H1′/H2′), 3.92 (s, 3H, H1′/H2′). HRMS-ESI(+) Calculated for C_25_H_16_NO_6_S_2_ [M + H]^+^ 490.0414; Found 490.0408.

*2-cyano-3-(5′-((5,7-dimethoxy-2-oxo-2H-chromen-3-yl)ethynyl)-[2,2′-bithiophen]-5-yl)acrylic acid* (**9b**). Starting from 23.2 mg (0.055 mmol, 1 eq.) of 5′-((5,7-dimethoxi-2-oxo-2*H*-chromen-3-yl)ethynyl)-[2,2′-bithiophene]-5-carbaldehyde (**5b**), 14.1 mg (52.5%) of 2-cyano-3-(5′-((5,7-dimethoxy-2-oxo-2*H*-chromen-3-yl)ethynyl)-[2,2′-bithiophen]-5-yl)acrylic acid (**9b**) were obtained. ^1^H-NMR (400 MHz, DMF-*d*_7_) δ (ppm) 8.53 (s, 1H, H4/H9′), 8.27 (s, 1H, H4/H9′), 8.04 (s, 1H, H5′/H6′/H7′/H8′), 7.70 (br s, 1H, H5′/H6′/H7′/H8′), 7.67 (br s, 1H, H5′/H6′/H7′/H8′), 7.52 (br s, 1H, H5′/H6′/H7′/H8′), 6.65 (s, 1H, H6/H8), 6.61 (s, 1H, H6/H8), 4.04 (s, 3H, H1′/H2′), 3.99 (s, 3H, H1′/H2′). HRMS-ESI(+) Calculated for C_25_H_16_NO_6_S_2_ [M + H]^+^ 490.0414; Found 490.0406.

*2-cyano-3-(5-((6,7-dimethoxy-2-oxo-2H-chromen-3-yl)ethynyl)-thiophen-2-yl)acrylic acid* (**10**). Starting from 28.1 mg (0.083 mmol, 1 eq.) of 5-((6,7-dimethoxy-2-oxo-2*H*-chromen-3-yl)ethynyl)-thiophene-2-carbaldehyde (**6**), 17.7 mg (50.8%) of 2-cyano-3-(5-((6,7-dimethoxy-2-oxo-2*H*-chromen-3-yl)ethynyl)-thiophen-2-yl)acrylic acid (**10**) were obtained. ^1^H-NMR (400 MHz, DMSO-*d*_6_) δ (ppm) 8.50 (s, 1H, H4/H7′), 8.36 (s, 1H, H4/H7′), 7.98 (d, *J* = 3.8 Hz, 1H, H5′/H6′), 7.57 (d, *J* = 3.9 Hz, 1H, H5′/H6′), 7.22 (s, 1H, H5/H8), 7.11 (s, 1H, H5/H8), 3.89 (s, 3H, H1′/H2′), 3.81 (s, 3H, H1′/H2′). HRMS-ESI(+) Calculated for C_21_H_14_NO_6_S [M + H]^+^ 408.0536; Found 408.0529.

*2-cyano-3-(2-decyl-7-((6,7-dimethoxy-2-oxo-2H-chromen-3-yl)ethynyl)-2H-benzo[d][1,2,3]triazol-4-yl)acrylic acid* (**11**). Starting from 51,7 mg (0.10 mmol, 1 eq.) of 2-decyl-7-((6,7-dimethoxy-2-oxo-2*H*-chromen-3-yl)ethynyl)-2*H*-benzo[*d*][1,2,3]triazole-4-carbaldehyde (**7**), 8.9 mg (50.8%) of 2-cyano-3-(2-decyl-7-((6,7-dimethoxy-2-oxo-2*H*-chromen-3-yl)ethynyl)-2*H*-benzo[*d*][1,2,3]triazol-4-yl)acrylic acid (11) were obtained. 1H-NMR (400 MHz, DMF-*d*_7_) δ (ppm) 8.92 (s, 1H, H4/H7′), 8.55 (d, *J* = 8.0 Hz, 1H, H5′/H6′), 8.46 (s, 1H, H4/H7′), 7.97 (d, *J* = 7.8 Hz, 1H, H5′/H6′), 7.42 (s, 1H, H5/H8), 7.17 (s, 1H, H5/H8), 4.95 (t, *J* = 7.3 Hz, 2H, H1′′), 4.04 (s, 3H, H1′/H2′), 3.93 (s, 3H, H1′/H2′), 2.22–2.15 (m, 2H, H2′′), 1.44–1.14 (m, 20H, H3′-H9′′), 0.85 (t, *J* = 6.4 Hz, 5H, H10′′). HRMS-ESI(+) Calculated for C_33_H_35_N_4_O_6_ [M + H]^+^ 583.2551; Found 583.2542.

### 3.3. Theoretical Calculations

The ground state molecular geometry was optimized using the density functional theory (DFT) by means of the Gaussian 09 program (Gaussian, Wallingford, CT, USA) [53], under CAM-B3LYP/6-311G(d,p) level [54,55] taking into account the bulk solvent effects of acetonitrile [56]. Optimal geometries were determined on isolated entities in acetonitrile and no conformation restrictions were imposed. For the resulting optimized geometries time-dependent DFT calculations (using the same functional and basis set as those in the previously calculations) were performed to predict the vertical electronic excitation energies. Molecular orbital contours were predicted at the B3LYP/6-311G(d,p) level of theory in vacuo and were plotted using GaussView 5.0.8 (Gaussian). The orbitals transitions percentage contributions of the predicted vertical excitation were calculated using GaussSum 2.2 (Dublin, Ireland) [57].

### 3.4. DSSCs Fabrication and Photovoltaic Characterization

The detailed procedure has been described elsewhere [58]. The conductive FTO-glass (TEC7, Greatcell Solar, Queanbeyan, Australia) used for the preparation of the transparent electrodes was first cleaned with detergent and then washed with water and ethanol. To prepare the anodes, the conductive glass plates (area: 15 cm × 4 cm) were immersed in a TiCl_4_/water solution (40 mM) at 70 °C for 30 min, washed with water and ethanol and sintered at 500 °C for 30 min. This procedure is essential to improve the adherence of the subsequently deposited nanocrystalline layers to the glass plates, as well as to serve as a ‘blocking-layer’, helping to block charge recombination between electrons in the FTO and holes in the I^−^/I_3_^−^ redox couple. Afterwards, the TiO_2_ nanocrystalline layers were deposited on these pre-treated FTO plates by screen-printing the transparent titania paste (18NR-T, Greatcell Solar) using a frame with polyester fibers with 43.80 mesh per cm^2^. This procedure, involving two steps (coating and drying at 125 °C), was repeated twice. The TiO_2_-coated plates were gradually heated up to 325 °C, then the temperature increased to 375 °C in 5 min, and afterwards to 500 °C. The plates were sintered at this temperature for 30 min, and finally cooled down to room temperature. A second treatment with the same TiCl_4_/water solution (40 mM) was performed, following the procedure described previously. This second TiCl_4_ treatment is also an optimization step that enhances the surface roughness for dye adsorption, thus positively affecting the photocurrent produced by the cell under illumination. Finally, a coating of reflective titania paste (WER2-O, Greatcell Solar) was deposited by screen-printing and sintered at 500 °C. This layer of 150–200 nm sized anatase particles functions as a ‘photon-trapping’ layer that further improves the photocurrent. Each anode was cut into rectangular pieces (area: 2 cm × 1.5 cm) with a spot area of 0.196 cm^2^ and a thickness of 15 μm. The prepared anodes were soaked for 16 h in a 0.5 mM solution of the dye in dichloromethane:methanol:H_2_O (65:20:2), at room temperature in the dark. The excess dye was removed by rinsing the photoanodes with the same solvent as that employed for the dye solution.

Each counter-electrode consisted of an FTO-glass plate (area: 2 cm × 2 cm) in which a hole (1.0 mm diameter) was drilled. The perforated substrates were washed and cleaned with water and ethanol to remove any residual glass powder and organic contaminants. The transparent Pt catalyst (PT1, Greatcell Solar) was deposited on the conductive face of the FTO-glass by doctor blade: one edge of the glass plate was covered with a strip of an adhesive tape (3 M Magic) both to control the thickness of the film and to mask an electric contact strip. The Pt paste was spread uniformly on the substrate by sliding a glass rod along the tape spacer. The adhesive tape strip was removed, and the glasses heated at 550 °C for 30 min. The photoanode and the Pt counter-electrode were assembled into a sandwich type arrangement and sealed (using a thermopress) with a hot melt gasket made of Surlyn ionomer (Meltonix 1170-25, Solaronix SA, Aubonne, Switzerland). The electrolyte was prepared by dissolving the redox couple, I^−^/I_3_^−^ (0.8 M LiI and 0.05 M I_2_), in an acetonitrile/valeronitrile (85:15, % *v*/*v*) mixture. The electrolyte was introduced into the cell via backfilling under vacuum through the hole drilled in the back of the cathode. Finally, the hole was sealed with adhesive tape.

For each compound, at least two cells were assembled under the same conditions, and the efficiencies were measured 5 times for each cell resulting in a minimum of 10 measurements per compound.

Current-Voltage curves were recorded with a digital Keithley SourceMeter multimeter (PVIV-1A) (Newport, M. T. Brandão, Porto, Portugal) connected to a PC. Simulated sunlight irradiation was provided by an Oriel solar simulator (Model LCS-100 Small Area Sol1A, 300 W Xe Arc lamp equipped with AM 1.5 filter, 100 mW/cm^2^) (Newport, M. T. Brandão). The thickness of the oxide film deposited on the photoanodes was measured using an Alpha-Step D600 Stylus Profiler (KLA-Tencor, Milpitas, CA, USA).

## 4. Conclusions

In the current work, five new 3-ethynylaryldimethoxycoumarin-based chromophores were synthesized by previously reported methods, with the aim of investigating not only the effect of varying the heterocyclic π-bridge on the device performance, but also assess the difference in properties between the 6,7- and 5,7-substitution patterns on the coumarin moiety. Through absorption and fluorescence emission spectroscopy, both in solution and adsorbed onto porous TiO_2_, it was determined that dyes **8** and **9b** present the most redshifted absorption, highest absorptivity, and most pronounced Stokes shift, which help explain their high photocurrent values. Additionally, the difference in the theoretically calculated HOMO orbitals of dyes **9a** and **9b** further support the more electron-donating nature of the 5,7-substitution pattern. Despite this, this set of dyes demonstrates comparatively lower conversion efficiencies than other coumarin dyes [26,27,28,29,30,31,32], which can be attributed to the superior donor ability of nitrogen-based donors there used. Overall, we can see that the increased planarity of the thieno [3,2-*b*] thiophene π-bridge (dye **8**) and the superior donor ability of the 5,7-disubstituted coumarin are promising avenues for the synthesis of more efficient coumarin-based donors.

## Figures and Tables

**Table 1:** Spectroscopic data for compounds **8**–**11** in acetonitrile solution (absorption and fluorescence emission maxima, molar extinction coefficients, ε, and Stokes shift, Δ_SS_) and absorbed in TiO_2_ films (absorption and fluorescence emission maxima) at 293 K.

Dye	$\lambda_{max}^{Abs}$ (mm)	$\lambda_{max}^{Abs}$ Solid (nm)	ε (cm^−1^M^−1^)	$\lambda_{max}^{Fluo}$ (nm)	$\lambda_{max}^{Fluo}$ Solid (nm)	Δ_SS_ (nm)	Δ_SS_ (cm^−1^)
**8**	417	386	64,470	489	476	72	3589
**9a**	420	380	30,190	464	510	44	2201
**9b**	418	380	45,480	499	500	81	3998
**10**	414	375	15,300	486	488	72	3578
**11**	416	375	12,060	462	490	46	2451

**Table 2:** Experimental absorption maxima obtained in acetonitrile solution together with the relevant computed absorption properties (predicted vertical excitation energies and associated orbitals transitions major contributions together with oscillator strengths, f, and band gap, E_g_) for the investigated compounds obtained by TD-DFT at the CAM-B3LYP/6-311G(d,p) level of theory after ground-state geometry optimization using the same functional and basis set.

Dye	$\lambda_{max}^{S_{0}\to S_{n}}\;(\mathrm{nm})$	$\lambda_{max}^{S_{0}\to S_{n}}\mathrm{Calc}.$ (nm)	Dipole Moment (D)	Transition and Orbitals Major Contributions	Oscillator Strength, F	E_g_ (eV) ^a^
**8**	416	415	14.560	S_0_→S_1_, HOMO→LUMO (83%)	2.146	2.85 [2.70]
**9a**	421	424	19.139	S_0_→S_1_, HOMO→LUMO (79%)	2.156	2.77 [2.75]
**9b**	416	428	22.394	S_0_→S_1_, HOMO→LUMO (78%);	2.147	2.72 [2.73]
**10**	414	404	24.230	S_0_→S_1_, HOMO→LUMO (86%)	1.878	2.97 [2.78]
**11**	415	407	15.56	S_0_→S_1_, HOMO→LUMO (84%)	1.764	2.75 [2.76]

^a^ in brackets the experimental absorption band gap obtained from the intersection between the normalized absorption and fluorescence emission spectra.

**Table 3:** Electrochemical properties in dimethylformamide (DMF) obtained from DPV measurements: HOMO energy level, determined from the onset of the oxidation peak (E_ox_); LUMO energy level, determined from the onset of the reduction peak (E_red_). Gap energy (E_g_), calculated with E_HOMO_—E_LUMO_.

Dye	HOMO Energy (eV)	LUMO Energy (eV)	E_g_ (eV)
**8**	−5.70	−3.57	2.13
**9a**	−5.64	−3.58	2.06
**9b**	−5.60	−3.53	2.07
**10**	−5.74	−3.62	2.12
**11**	−5.65	−3.60	2.05

**Table 4:** Performance values of the test cells based on the synthesized dyes and reference dye N719 under 100 mW·cm^−2^ AM 1.5 illumination. The results presented correspond to the average values of at least two cells per dye, each cell measured 5 times.

Dye	V_oc_ (mV)	J_sc_ (mA/cm^2^)	J_max_ (mA/cm^2^)	V_max_ (mV)	FF	η (%)
**8**	367 ± 5	9.3 ± 0.1	7.5 ± 0.2	256 ± 3	0.56 ± 0.01	2.00 ± 0.06
**9a**	289 ± 6	6.7 ± 0.3	4.9 ± 0.3	193 ± 4	0.49 ± 0.02	0.95 ± 0.07
**9b**	339 ± 3	10.2 ± 0.1	7.8 ± 0.2	227 ± 2	0.51 ± 0.01	1.78 ± 0.06
**10**	311 ± 5	6.4 ± 0.1	4.9 ± 0.3	214 ± 5	0.54 ± 0.02	1.07 ± 0.05
**11**	359 ± 2	5.4 ± 0.1	4.3 ± 0.2	258 ± 1	0.58 ± 0.02	1.13 ± 0.04
**N719**	440 ± 6	15.5 ± 0.4	13.1 ± 0.2	305 ± 4	0.59 ± 0.02	4.06 ± 0.05

## Data Availability

The data provided in this study is available in the article and Supplementary Material file submitted.

## Supplementary Materials

The following are available online, the detailed synthetic procedures, the NMR and HRMS spectral data and the full differential pulse voltammograms of dyes **8**–**11**.

## Sample Availability

Samples of the compounds are not available from the authors.
