# Supplementary material for: New 3-Ethynylaryl Coumarin-Based Dyes for DSSC Applications: Synthesis, Spectroscopic Properties, and Theoretical Calculations
Source: Molecules. 2021 May 14;26(10):2934. doi: 10.3390/molecules26102934 (PMC8156968; doi:10.3390/molecules26102934)

Supporting Information

New 3-Ethynylaryl Coumarin-Based Dyes for DSSC Applications: Synthesis, spectroscopic properties and theoretical calculations

João Sarrato[1,†], Ana Lucia Pinto[1,†], Gabriela Malta[1,†], Eva Röck[3], João Pina[2], A. Jorge Parola[1,*], J. Carlos Lima[1] and Paula S. Branco[1,*]

[1]LAQV-REQUIMTE, Departament of Chemistry, NOVA School of Science and Technology, FCT NOVA, Universidade NOVA de Lisboa, 2829-516, Caparica, Portugal; j.sarrato@campus.fct.unl.pt (J.S.); al.pinto@campus.fct.unl.pt (A.L.P.); g.malta@campus.fct.unl.pt (G.M); lima@fct.unl.pt (J.C.L.)

[2]University of Coimbra, Coimbra Chemistry Centre, Department of Chemistry, Rua Larga, 3004-535 Coimbra, Portugal. jpina@qui.uc.pt (J.P.)

[3]University of Bern, Department of Chemistry, Biochemistry and Pharmaceutical Sciences, Freiestrasse 3, CH-3012 Bern, Switzerland. eva.roeck@dcb.unibe.ch (E.R.)

* Correspondence: ajp@fct.unl.pt (A.J.P.); paula.branco@fct.unl.pt (P.B.); Tel.: (+351) 21 294 8300

† These authors contributed equally to this work.

Contents

1 Synthesis of 3-ethynylcoumarins S4

1.1 Synthesis of 5,7-diethylcarbonatecoumarin S4

1.2 Synthesis of 3-bromo-5,7-dihydroxycoumarin S4

1.3 Synthesis of 3-bromo-6,7-dimethoxycoumarin (**1a**) S5

1.4 Synthesis of 6,7-dimethoxy-3-((trimethylsilyl)ethynyl)coumarin (**2a**) and 5,7-dimethoxy-3-((trimethylsilyl)ethynyl)coumarin (**2b**) S5

1.5 Synthesis of 6,7-dimethoxy-3-ethynylcoumarin (**3a**) and 5,7-dimethoxy-3-ethynylcoumarin (**3b**) S6

2 Synthesis of aromatic aldehydes S7

2.1 Synthesis of 5-bromothieno-[3,2-*b*]-tiophene-2-carbaldehyde S7

2.2 Synthesis of 2-decyl-*2H*-benzo[*d*][1,2,3]triazole S7

2.3 Synthesis of 4,7-dibromo-2-decyl-*2H*-benzo[*d*][1,2,3]triazole S8

2.4 Synthesis of 7-dibromo-2-decyl-2H-benzo[d][1,2,3]triazole-4-carbaldehyde S8

3 Dye synthesis S9

3.1 General method for the synthesis of coupled aldehydes (**4** - **7**) S9

3.2 General method for the synthesis of final chromophores (**8** - **11**) S11

4 Spectra S15

^{1}H-NMR (400 MHz, $CDCl_3$) spectrum of diethyl (2-oxo-2H-chromene-5,7-diyl) bis(carbonate) S15

^{1}H-NMR (400 MHz, $CDCl_3$) spectrum of 3-bromo-5,7-dihydroxycoumarin S15

^{1}H-NMR (400 MHz, $CDCl_3$) spectrum of 3-bromo-6,7-dihydroxycoumarin (**1a**) S16

^{1}H-NMR (400 MHz, $CDCl_3$) spectrum of 6,7-dimethoxy-3-((trimethylsilyl)ethynyl)coumarin (**2a**) S16

^{13}C-NMR (101 MHz, $CDCl_3$) spectrum of 6,7-dimethoxy-3-((trimethylsilyl)ethynyl)coumarin (**2a**) S17

HRMS-ESI spectrum of 6,7-dimethoxy-3-((trimethylsilyl)ethynyl)coumarin (**2a**) S17

^{1}H-NMR (400 MHz, $CDCl_3$) spectrum of 5,7-dimethoxy-3-((trimethylsilyl)ethynyl)coumarin (**2b**) S18

HRMS-ESI spectrum of 5,7-dimethoxy-3-((trimethylsilyl)ethynyl)coumarin (**2b**) S18

^{1}H-NMR (400 MHz, $CDCl_3$) spectrum of 5-bromothieno-[3,2-*b*]-tiophene-2-carbaldehyde . S19

Mass spectra of 5-bromothieno-[3,2-*b*]-tiophene-2-carbaldehyde S19

^{1}H-NMR (400 MHz, $CDCl_3$) spectrum of 2-decyl-*2H*-benzo[*d*][1,2,3]triazole S20

^{1}H-NMR (400 MHz, $CDCl_3$) spectrum of 4,7-dibromo-2-decyl-*2H*-benzo[*d*][1,2,3]triazole... S20

^{1}H-NMR (400 MHz, $CDCl_3$) spectrum of 7-dibromo-2-decyl-2H-benzo[d][1,2,3]triazole-4-carbaldehyde S21

^{1}H-NMR (400 MHz, $CDCl_3$) spectrum of 5-((6,7-dimethoxy-2-oxo-2H-chromen-3-yl)ethynyl)thieno[3,2-b]tiophene-2-carbaldehyde (**4**) S21

HRMS-ESI spectrum of 5-((6,7-dimethoxy-2-oxo-2H-chromen-3-yl)ethynyl)thieno[3,2-b]tiophene-2-carbaldehyde (**4**) S22

^{1}H-NMR (400 MHz, CD_2Cl_2) spectrum of 5’-((6,7-dimethoxi-2-oxo-2H-chromen-3-yl)ethynyl)-[2,2’-bithiophene]-5-carbaldehyde (**5a**) S22

HRMS-ESI spectrum of 5’-((6,7-dimethoxi-2-oxo-2H-chromen-3-yl)ethynyl)-[2,2’-bithiophene]-5-carbaldehyde (**5a**) S23

^{1}H-NMR (400 MHz, DMF-d_7) spectrum of 5’-((5,7-dimethoxi-2-oxo-2H-chromen-3-yl)ethynyl)-[2,2’-bithiophene]-5-carbaldehyde (**5b**) S23

HRMS-ESI spectrum of 5’-((5,7-dimethoxi-2-oxo-2H-chromen-3-yl)ethynyl)-[2,2’-bithiophene]-5-carbaldehyde (**5b**) S24

^{1}H-NMR (400 MHz, CD_2Cl_2) spectrum of 5-((6,7-dimethoxi-2-oxo-2H-chromen-3-yl)ethynyl)-thiophene-5-carbaldehyde (**6**) S24

HRMS-ESI spectrum of 5-((6,7-dimethoxi-2-oxo-2H-chromen-3-yl)ethynyl)-thiophene-5-carbaldehyde (**6**) S25

^{1}H-NMR (400 MHz, CD_2Cl_2) spectrum of 2-decyl-7-((6,7-dimethoxy-2-oxo-2H-chromen-3-yl)ethynyl)-2H-benzo[d][1,2,3]triazole-4-carbaldehyde (**7**) S25

HRMS-ESI spectrum of 2-decyl-7-((6,7-dimethoxy-2-oxo-2H-chromen-3-yl)ethynyl)-2H-benzo[d][1,2,3]triazole-4-carbaldehyde (**7**) S26

^{1}H-NMR (400 MHz, DMSO-d_6) spectrum of 2-cyano-3-(5-((6,7-dimethoxy-2-oxo-2H-chromen-3-yl)ethynyl)thieno[3,2-b]thiophen-2-yl)acrylic acid (**8**) S26

HRMS-ESI spectrum of 2-cyano-3-(5-((6,7-dimethoxy-2-oxo-2H-chromen-3-yl)ethynyl)thieno[3,2-b]thiophen-2-yl)acrylic acid (**8**) S27

^{1}H-NMR (400 MHz, DMF-d_7) spectrum of 2-cyano-3-(5’-((6,7-dimethoxy-2-oxo-2H-chromen-3-yl)ethynyl)-[2,2’-bithiophen]-5-yl)acrylic acid (**9a**) S27

HRMS-ESI spectrum of 2-cyano-3-(5’-((6,7-dimethoxy-2-oxo-2H-chromen-3-yl)ethynyl)-[2,2’-bithiophen]-5-yl)acrylic acid (**9a**) S28

^{1}H-NMR (400 MHz, DMF-d_7) spectrum of 2-cyano-3-(5’-((5,7-dimethoxy-2-oxo-2H-chromen-3-yl)ethynyl)-[2,2’-bithiophen]-5-yl)acrylic acid (**9b**) S28

HRMS-ESI spectrum of 2-cyano-3-(5’-((5,7-dimethoxy-2-oxo-2H-chromen-3-yl)ethynyl)-[2,2’-bithiophen]-5-yl)acrylic acid (**9b**) S29

^{1}H-NMR (400 MHz, DMSO-d_6) spectrum of 2-cyano-3-(5-((6,7-dimethoxy-2-oxo-2H-chromen-3-yl)ethynyl)-thiophen-2-yl)acrylic acid (**10**) S29

HRMS-ESI spectrum of 2-cyano-3-(5-((6,7-dimethoxy-2-oxo-2H-chromen-3-yl)ethynyl)-thiophen-2-yl)acrylic acid (**10**) S30

^{1}H-NMR (400 MHz, DMF-d_7) spectrum of 2-cyano-3-(2-decyl-7-((6,7-dimethoxy-2-oxo-2H-chromen-3-yl)ethynyl)-2H-benzo[d][1,2,3]triazol-4-yl)acrylic acid (**11**) S30

HRMS-ESI spectrum of 2-cyano-3-(2-decyl-7-((6,7-dimethoxy-2-oxo-2H-chromen-3-yl)ethynyl)-2H-benzo[d][1,2,3]triazol-4-yl)acrylic acid (**11**) S31

1 Synthesis of 3-ethynylcoumarins

1.1 Synthesis of 5,7-diethylcarbonatecoumarin

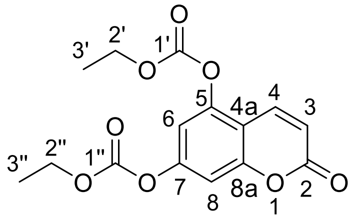

To a solution of 5,7-dihydroxycoumarin (659.1 mg, 3.7 mmol) in 5 mL of dry dioxane, 0.72 mL of pyridine (8.9 mmol, 2.4 eq.) and 0.85 mL of ethyl chloroformate (8.9 mmol, 2.4 eq.) were added at 0°C. The reaction was stirred at room temperature for 70 h under nitrogen atmosphere. The solvent was removed, and the product was extracted with dichloromethane. The organic layers were combined, dried with anhydrous Na_2SO_4, filtered and dried under reduced atmosphere. The crude was purified by flash column chromatography with hexane/AcOEt (1:1) as eluent to give 748.5 mg (63 %) of 5,7-diethylcarbonatecoumarin.

^{1}H NMR (400 MHz, $CDCl_3$) δ (ppm) 7.84 (d, *J* = 10.0 Hz, 1H, H4), 7.19 (d, *J* = 2.4, 1H, H6/H8), 7.14 (d, *J* = 2.0 Hz, 1H, H6/H8), 6.41 (d, *J* = 9.6 Hz, 1H, H3), 4.36 (m, 4H, H2’), 1.41 (m, 6H, H3’).

1.2 Synthesis of 3-bromo-5,7-dihydroxycoumarin

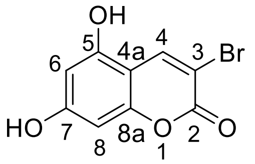

To a solution of 3-Bromo-2-oxo-2H-chromene-5,7-diyl diethyl bis(carbonate) (105.4mg, 0.26 mmol) in 2.2 mL of THF, 20 mL of a saturated aqueous solution of $NaHCO_3$ were added. After 20h the reaction was complete, the solvent was removed and the product was extracted once with Et_2O, then the aqueous phase was acidified with an aqueous solution of HCl 1M and the product was extracted with dichloromethane. The combined organic layers were dried with anhydrous Na_2SO_4, filtered and evaporated. The obtained crude containing 3-bromo-5,7-dihydroxicoumarin (100.6 mg) was methylated directly without further purification.

1.3 Synthesis of 3-bromo-6,7-dimethoxycoumarin (**1a**)

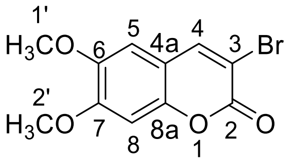

To a round-bottom flask containing 277 mg (1.34 mmol) of 6,7-dimethoxycoumarin 423 mg (1.38 mmol, 1 eq.) of Oxone®, 1.5 mL (2.96 mmol, 2.2 eq.) of a 2M solution of HBr and 10 mL de dichloromethane were added and the resulting solution was stirred at room temperature for 28h. The progress of the reaction was monitored by TLC (hexane/AcOEt (1:1 v/v)) and, once finished, distilled H_2O was added and the solution was extracted 4x with DCM. The combined organic layers were then dried over anhydrous Na_2SO_4, filtered and evaporated to dryness under reduced pressure, affording 377.8 mg (98.7%) of 3-bromo-6,7-dimethoxycoumarin (**1a**) as a beige solid.

^{1}H NMR (400 MHz, $CDCl_3$) δ (ppm) 8.00 (s, 1H, H4), 6.84 (s, 1H, H5), 6.81 (s, 1H, H8), 3.95 (s, 3H, H1'/H2'), 3.92 (s, 3H, H1'/H2').

1.4 Synthesis of 6,7-dimethoxy-3-((trimethylsilyl)ethynyl)coumarin (**2a**) and 5,7-dimethoxy-3-((trimethylsilyl)ethynyl)coumarin (**2b**)

To a sealed tube, 0.06 eq. of PPh_3, 0.12 eq. of CuI, 0.15 eq. of $Pd(PPh_3)_4$, 1 eq. of 3-bromocoumarin (**1a**/**1b**) and 5 mL of dry dioxane were added under a N_2 atmosphere. After a few minutes, 2 eq. of ethynyltrimethylsilane and 0.35 mL 2 eq. of dry (*i-Pr*)$_2$NH were added and the solution was stirred at 45°C overnight under a N_2 atmosphere. Once the reaction was confirmed to be complete by TLC (hexane/AcOEt (7:3 v/v)), the solution was cooled to room temperature, the solvent was removed under reduced pressure and the solid residue dried *in vacuo* before being purified by flash chromatography with hexane/AcOEt (7:3 v/v) as eluent, affording the target compounds.

6,7-dimethoxy-3-((trimethylsilyl)ethynyl)coumarin (**2a**)

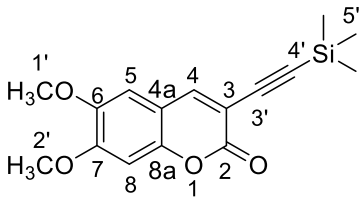

Starting from 347.3 mg (1.22 mmol, 1 eq.) of 3-bromo-6,7-dimethoxycoumarin (**1a**), 343.4 mg (93.2%) of 6,7-dimethoxy-3-((trimethylsilyl)ethynyl)coumarin (**2a**) were obtained.

^{1}H NMR (400 MHz, $CDCl_3$) δ (ppm) 7.83 (s, 1H, H4), 6.82 (s, 1H, H5/H8), 6.80 (s, 1H, H5/H8), 3.95 (s, 3H, H1'/H2'), 3.91 (s, 3H, H1'/H2'), 0.26 (s, 9H, H5').

^{13}C NMR (101 MHz, $CDCl_3$) δ (ppm) 160.0 (C2), 153.5 (C7), 149.8 (C8a), 146.8 (C4/C6), 146.1 (C4/C6), 111.5 (C3/C4a/C5), 109.5 (C3/C4a/C5), 107.7 (C3/C4a/C5), 101.0 (C3'/C4'/C8), 99.9 (C3'/C4'/C8), 98.7 (C3'/C4'/C8), 56.6 (C1'/C2'), 56.5 (C1'/C2'), -0.1 (C5').

HRMS-ESI(+) Calculated for $C_{16}H_{19}O_4Si$ $[M+H]^+$ 303.1047; Found 303.1053

5,7-dimethoxy-3-((trimethylsilyl)ethynyl)coumarin (**2b**)

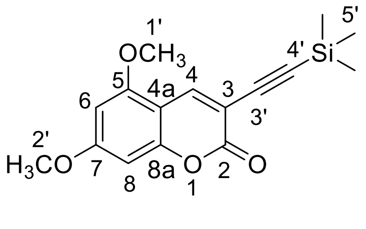

Starting from 147.1 mg (0.52 mmol, 1 eq.) of 3-bromo-5,7-dimethoxycoumarin (**1b**), 114.0 mg (72.9%) of 5,7-dimethoxy-3-((trimethylsilyl)ethynyl)coumarin (**2b**) were obtained.

^{1}H NMR (400 MHz, $CDCl_3$) δ (ppm) 8.14 (s, 1H, H4), 6.37 (s, 1H, H6/H8), 6.25 (d, J = 2.4 Hz, 1H, H6/H8), 3.88 (s, 3H, H1'/H2'), 3.84 (s, 3H, H1'/H2'), 0.25 (s, 9H, H5').

HRMS-ESI(+) Calculated for $C_{16}H_{19}O_4Si$ $[M+H]^+$ 303.1047; Found 303.1041

1.5 Synthesis of 6,7-dimethoxy-3-ethynylcoumarin (3a) and 5,7-dimethoxy-3-ethynylcoumarin (3b)

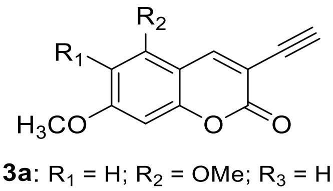

3a: R_1 = H; R_2 = OMe; R_3 = H
3b: R_2 = H; R_1 = OMe; R_3 = H

To a round-bottom flask containing 102.6 mg (0.34 mmol) of dimethoxy-3-((trimethylsilyl)ethynyl)coumarin (**2a/2b**), 6 mg (0.044 mmol, 0.13 eq.) of K_2CO_3 and 5 ml of dry MeOH were added. After approximately 4h-4:30h of stirring at room temperature, the conclusion of the reaction was confirmed by TLC (hexane/AcOEt (6:4 v/v)), the solution was evaporated to dryness and the product was used directly in the following Sonogashira reaction without further purification.

2 Synthesis of aromatic aldehydes

2.1 Synthesis of 5-bromothieno-[3,2-*b*]-tiophene-2-carbaldehyde

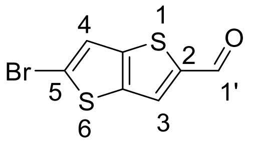

A round-bottom flask containing 200.2 mg (0.671 mmol) of 2,5-dibromothieno-[3,2-*b*]thiophene in 5 mL of dry THF was cooled in an acetone/liquid N_2 bath until approximately -78°C and, after a few minutes, 0.47 mL (0.752 mmol, 1.1 eq.) of a 1,6M solution of *n*-BuLi in hexanes were added dropwise. After stirring for 15 minutes at -78°C, 52 µL (0.671 mmol, 1 eq.) of dry DMF were added and the solution remained stirring under N_2 atmosphere at the same temperature for 3h. Once the total consumption of the starting material was confirmed by TLC (hexane/DCM (8:2 v/v)), the solution was slowly warmed to room temperature, distilled H_2O was added and the solution was extracted twice with AcOEt. The combined organic layers were washed with brine, dried over anhydrous Na_2SO_4, filtered and evaporated to dryness. The solid residue was purified by standard column chromatography with hexane/DCM (7:3 v/v) as eluent, affording 106.5 mg (64.2%) of 5-bromothieno-[3,2-b]-tiophene-2-carbaldehyde as a crystalline bege solid.

^{1}H NMR (400 MHz, $CDCl_3$) δ (ppm) 9.96 (s, 1H, H1'), 7.84 (s, 1H, H3/H4), 7.35 (s, 1H, H3/H4).

2.2 Synthesis of 2-decyl-*2H*-benzo[*d*][1,2,3]triazole

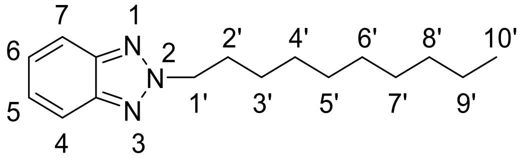

To a round-bottom flask containing 1.01 g (8.50 mmol) of benzotriazole, 3.60 mg (26.05, 3 eq.) of K_2CO_3, 6 mL of dry DMF and 2.18 mL (10.20 mmol, 1.2 eq.) of $C_{10}H_{21}I$ were added and the solution was stirred at room temperature for 2h. Once confirmed to be complete by TLC (hexane/AcOEt (6:4 v/v)), distilled H_2O was added and the solution was extracted 4x with DCM. The combined organic layers were washed 4x with distilled water, 1x with brine, dried over anhydrous Na_2SO_4, filtered and evaporated to dryness. The slid residue was purified by standard column chromatography with hexane/AcOEt (9:1 v/v) as eluent, affording 862.4 mg (39.8%) of 2-decyl-2H-benzo[d][1,2,3]triazole as a yellowish oil.

^{1}**H NMR** (400 MHz, $CDCl_3$) δ (ppm) 7.86 (m, 2H, H4/H7), 7.37 (m, 2H, H5/H6), 4.72 (t, *J* = 7.2 Hz, 2H, H1'), 2.12 (t, *J* = 7.2 Hz, 2H, H2'), 1.38 - 1.19 (m, 15H, H3'-H9'), 0.87 (t, *J* = 6.6 Hz, 3H, H10').

2.3 Synthesis of 4,7-dibromo-2-decyl-*2H*-benzo[*d*][1,2,3]triazole

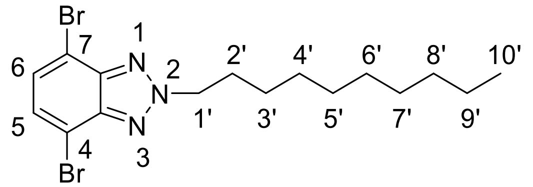

To a two-necked round-bottom flask containing 862 mg (3.33 mmol) of 2-decyl-2H-benzo[d][1,2,3]triazole, 4 mL of a 5.8 M solution of HBr (23.2 mmol, 7 eq.) were added and the solution was stirred at 100°C for 1h, after which 0.51 (9.97 mmol, 3 eq.) mL of Br_2 were added. After stirring at 135-140°C for 18h, the conclusion of the reaction was confirmed by TLC (DCM/hexane (8:2 v/v)), the solution was cooled to room temperature and distilled H_2O was added. The solution was extracted 3x with DCM, dried over anhydrous Na_2SO_4, filtered and evaporated to dryness. The solid residue was purified by flash column chromatography with hexane/DCM (7:3 v/v) as eluent, affording 598 mg (43.1%) of 4,7-dibromo-2-decyl-2H-benzo[d][1,2,3]triazole as a brown oil.

^{1}**H NMR** (400 MHz, $CDCl_3$) δ (ppm) 7,44 (s, 2H, H5/H6), 4,77 (t, *J* = 7.5 Hz, 2H, H1'), 2,19 - 2,10 (m, 2H, H2'), 1,41 - 1,21 (m, 19H, H3'-H9'), 0,87 (t, *J* = 6.7 Hz, 3H, H10').

2.4 Synthesis of 7-dibromo-2-decyl-2H-benzo[d][1,2,3]triazole-4-carbaldehyde

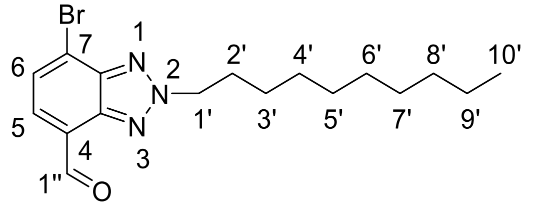

A two-necked round-bottom flask containing 362 mg (0.864 mmol) of 4,7-dibromo-2-decyl-*2H*-benzo[*d*][1,2,3]triazole in 5 mL of dry THF was cooled in an acetone/liquid N_2 bath until approximately -78°C and, after a few minutes, 0.6 mL (0.96 mmol, 1.1 eq.) of a 1,6M solution of *n*-BuLi in hexanes were added dropwise. After stirring for 15 minutes at -78°C, 70 µL (0.90 mmol, 1 eq.) of dry DMF were added and the solution remained stirring under N_2 atmosphere at the same temperature for 3h. Once the total consumption of the starting material was confirmed by TLC (hexane/AcOEt (9:1 v/v)), the solution was slowly warmed to room temperature, distilled H_2O was added and the solution was extracted twice with DCM. The combined organic layers dried over

anhydrous Na_2SO_4, filtered and evaporated to dryness. The solid residue was purified by standard column chromatography with hexane/AcOEt (9:1 v/v) as eluent, affording 95.9 mg (30.3%) of 7-dibromo-2-decyl-*2H*-benzo[*d*][1,2,3]triazole-4-carbaldehyde as a bege solid.

^{1}H NMR (400 MHz, $CDCl_3$) δ (ppm) 10.44 (s, 1H, H1''), 7.83 (d, *J* = 7.5 Hz, 1H, H5/H6), 7.76 (d, *J* = 7.6 Hz, 1H, H5/H6), 4.84 (t, *J* = 7.4 Hz, 3H, H1'), 2.17 (t, *J* = 7.3 Hz, 3H, H2'), 1.42 - 1.20 (m, 20H, H3'-H9'), 0.87 (t, *J* = 6.7 Hz, 4H, H10').

3 Dye synthesis

3.1 General method for the synthesis of coupled aldehydes (**4** - **7**)

To a sealed tube, PPh_3 (0.06 eq), CuI (0.12 eq), $Pd(PPh_3)_4$ (0.15), aldehyde (1 eq.) and 5 mL of dry dioxane were added under a N_2 atmosphere. After a few minutes ethynylcoumarin (**3**) (1 eq.) and dry (*i-Pr*)$_2$NH (2 eq.) were added and the solution was stirred at 45°C overnight under a N_2 atmosphere. Once the reaction was confirmed to be complete by TLC (hexane/AcOEt (7:3 v/v)), the solution was cooled to room temperature, the solvent was removed under reduced pressure and the solid residue dried *in vacuo* before being purified by flash chromatography with DCM/MeOH (99.8:0.02 v/v and 99.5:0.05 v/v) as eluent, affording a bright yellow solid in all cases.

5-((6,7-dimethoxy-2-oxo-*2H*-chromen-3-yl)ethynyl)thieno[3,2-*b*]tiophene-2-carbaldehyde (**4**):

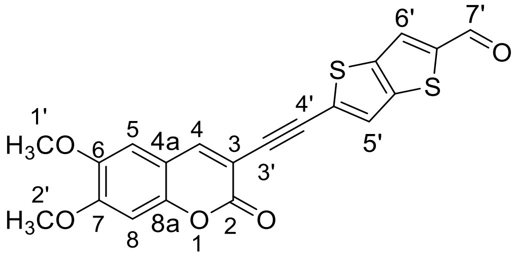

Starting from 104.6 mg (0.42 mmol, 1 eq.) of 5-bromothieno[3,2-*b*]thiophene-2-carbaldehyde (**C**), 89.5 mg (51.5%) of 5-((6,7-dimethoxy-2-oxo-*2H*-chromen-3-yl)ethynyl)thieno[3,2-*b*]tiophene-2-carbaldehyde (**4**) were obtained.

^{1}H NMR (400 MHz, $CDCl_3$) δ (ppm) 9.99 (s, 1H, H7'), 7.92 (s, 1H, H4), 7.89 (s, 1H, H5'/H6'), 7,54 (s, 1H, H5'/H6'), 6.87 (s, 1H, H5/H8), 6.86 (s, 1H, H5/H8), 3.98 (s, 3H, H1'/H2'), 3.94 (s, 3H, H1'/H2').

HRMS-ESI(+) Calculated for $C_{20}H_{13}O_5S_2$ $[M+H]^+$ 397.0199; Found 397.0197

5′-((6,7-dimethoxi-2-oxo-*2H*-chromen-3-yl)ethynyl)-[2,2′-bithiophene]-5-carbaldehyde (**5a**):

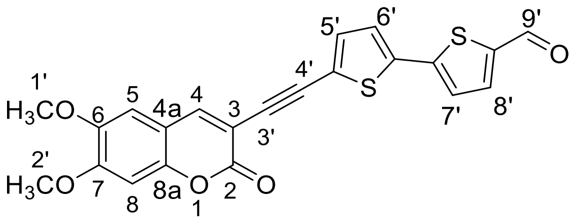

Starting from 132.8 mg (0.49 mmol, 1 eq.) of 5-bromo-[2,2′-bithiophene]-5-carbaldehyde, 95.2 mg (46.3%) of 5′-((6,7-dimethoxi-2-oxo-*2H*-chromen-3-yl)ethynyl)-[2,2′-bithiophene]-5-carbaldehyde (**5a**) were obtained.

^{1}H NMR (400 MHz, CD_2Cl_2) δ (ppm) 9.85 (s, 1H, H9′), 7.89 (s, 1H, H4), 7.71 (d, J = 4.8 Hz, 1H, H5′/H6′/H7′/H8′), 7.30 (s, 3H, H5′/H6′/H7′/H8′), 6.87 (s, 1H, H5/H8), 6.85 (s, 1H, H5/H8), 3.92 (s, 3H, H1′/H2′), 3.87 (s, 3H, H1′/H2′).

HRMS-ESI(+) Calculated for $C_{22}H_{15}O_5S_2$ $[M+H]^+$ 423.0355; Found 423.0348

5′-((5,7-dimethoxi-2-oxo-*2H*-chromen-3-yl)ethynyl)-[2,2′-bithiophene]-5-carbaldehyde (**5b**):

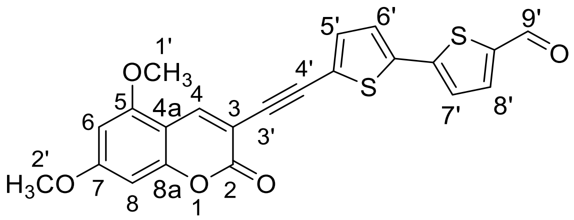

Starting from 154.0 mg (0.56 mmol, 1 eq.) of 5-bromo-[2,2′-bithiophene]-5-carbaldehyde, 22.4 mg (14.1%) of 5′-((5,7-dimethoxi-2-oxo-*2H*-chromen-3-yl)ethynyl)-[2,2′-bithiophene]-5-carbaldehyde (**5b**) were obtained.

^{1}H NMR (400 MHz, DMF-d_7) δ (ppm) 10.02 (s, 1H, H9′), 8.29 (s, 1H, H4), 8.08 (d, J = 4.03 Hz, 1H, H5′/H6′/H7′/H8′), 7.69 - 7,67 (m, 2H, H5′/H6′/H7′/H8′), 7,53 (d, J = 4.03 Hz 1H, H5′/H6′/H7′/H8′), 6.67 (s, 1H, H6/H8), 6.63 (s, 1H, H6/H8), 4.04 (s, 3H, H1′/H2′), 3.99 (s, 3H, H1′/H2′).

HRMS-ESI(+) Calculated for $C_{22}H_{15}O_5S_2$ $[M+H]^+$ 423.0355; Found 423.0349

5-((6,7-dimethoxy-2-oxo-*2H*-chromen-3-yl)ethynyl)-thiophene-2-carbaldehyde (**6**):

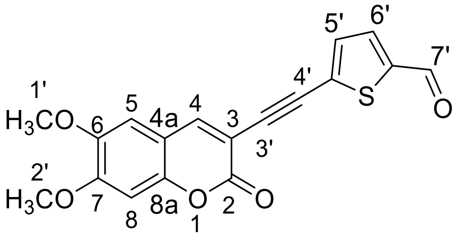

Starting from 64.8 mg (0.34 mmol, 1 eq.) of 5-bromothiophene-2-carbaldehyde, 58.7 mg (50.8%) of 5-((6,7-dimethoxy-2-oxo-*2H*-chromen-3-yl)ethynyl)-thiophene-2-carbaldehyde (**6**) were obtained.

^{1}H NMR (400 MHz, CD_2Cl_2) δ (ppm) 9.87 (s, 1H, H7'), 7.95 (s, 1H, H4), 7.71 (d, J = 3.8 Hz, 1H, H5'/H6'), 7.41 (d, J = 3.7 Hz, 1H, H5'/H6'), 6.89 (s, 1H, H5/H8), 6.87 (s, 1H, H5/H8), 3.94 (s, 3H, H1'/H2'), 3.89 (s, 3H, H1'/H2').

HRMS-ESI(+) Calculated for $C_{18}H_{13}O_5S$ $[M+H]^+$ 341.0478; Found 341.0478

2-decyl-7-((6,7-dimethoxy-2-oxo-*2H*-chromen-3-yl)ethynyl)-*2H*-benzo[*d*][1,2,3]triazole-4-carbaldehyde (**7**):

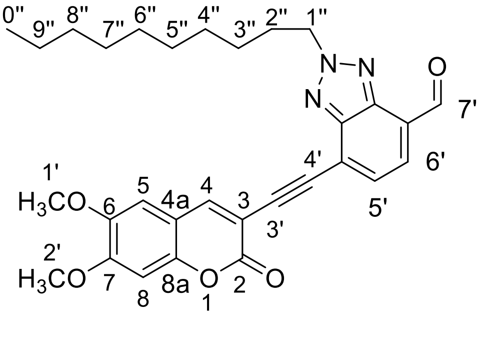

Starting from 113 mg (0.31 mmol, 1 eq.) of 7-dibromo-2-decyl-*2H*-benzo[*d*][1,2,3]triazole-4-carbaldehyde (**F**), 42.5 mg (50.8%) of 2-decyl-7-((6,7-dimethoxy-2-oxo-2H-chromen-3-yl)ethynyl)-2H-benzo[d][1,2,3]triazole-4-carbaldehyde (**7**) were obtained.

^{1}H NMR (400 MHz, CD_2Cl_2) δ (ppm) 10.50 (s, 1H, H7'), 8.11 (s, 1H, H4), 7.99 (d, J = 7.3 Hz, 1H, H5'/H6'), 7.81 (d, J = 7.5 Hz, 1H, H5'/H6'), 6.96 (s, 1H, H5/H8), 6.93 (s, 1H, H5/H8), 4.90 (t, J = 7.4 Hz, 2H, H1''), 3.99 (s, 3H, H1'/H2'), 3.94 (s, 3H, H1'/H2'), 2.26 - 2.18 (m, 2H, H2''), 1.43 - 1.30 (m, 15H, H3''-H9''), 0.90 (t, J = 6.4 Hz, 3H, H10'').

HRMS-ESI(+) Calculated for $C_{30}H_{34}N_3O_5$ $[M+H]^+$ 516.2493; Found 516.2509

3.2 General method for the synthesis of final chromophores (8 - 11)

To a round-bottom flask containing aldehyde (1 eq.), cyanoacetic acid (3 eq.), 5 mL of ACN and dry piperidine (2.7 eq.) were added and the resulting solution was stirred under reflux for 24h. Once the reaction was confirmed to be complete by TLC (DCM/MeOH (9.5:0.5 v/v)), the solvent was evaporated under reduced pressure, the solid residue was washed 3 - 5 times with ACN, acidified with HCl (10%) and washed 3 - 5 times with distilled water. After each washing step the solvent used was centrifuged (4500 rpm, 10-30 minutes) to recover any lost product.

2-cyano-3-(5-((6,7-dimethoxy-2-oxo-*2H*-chromen-3-yl)ethynyl)thieno[3,2-*b*]thiophen-2-yl)acrylic acid (**8**):

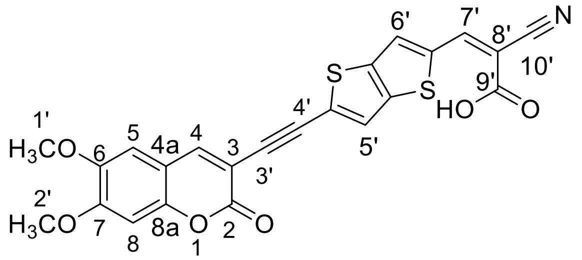

Starting from 15 mg (0.038 mmol, 1 eq.) of 5-((6,7-dimethoxy-2-oxo-*2H*-chromen-3-yl)ethynyl)thieno[3,2-*b*]tiophene-2-carbaldehyde (**4**), 7.8 mg (44.5%) of 2-cyano-3-(5-((6,7-dimethoxy-2-oxo-*2H*-chromen-3-yl)ethynyl)thieno[3,2-*b*]thiophen-2-yl)acrylic acid (**8**) were obtained.

^{1}H NMR (400 MHz, DMSO-*d_6*) δ (ppm) 8.75 (br s, 1H, OH), 8.37 (s, 1H, H4/H7'), 8.28 (s, 1H, H4/H7'), 8.09 (s, 1H, H5'/H6'), 7.89 (s, 1H, H5'/H6'), 7.26 (s, 1H, H5/H8), 7.14 (s, 1H, H5/H8), 3.90 (s, 3H, H1'/H2'), 3.82 (s, 3H, H1'/H2').

HRMS-ESI(+) Calculated for $C_{23}H_{14}NO_6S_2$ $[M+H]^+$ 464.0257; Found 464.0249

2-cyano-3-(5'-((6,7-dimethoxy-2-oxo-*2H*-chromen-3-yl)ethynyl)-[2,2'-bithiophen]-5-yl)acrylic acid (**9a**):

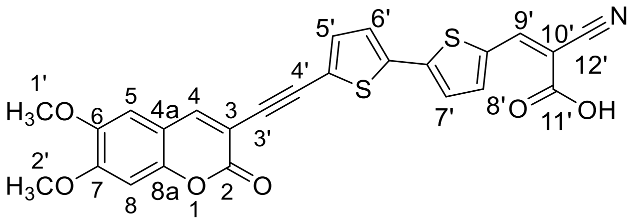

Starting from 95 mg (0.225 mmol, 1 eq.) of 5'-((6,7-dimethoxi-2-oxo-*2H*-chromen-3-yl)ethynyl)-[2,2'-bithiophene]-5-carbaldehyde (**5a**), 54.7 mg (49.7%) of 2-cyano-3-(5'-((6,7-dimethoxy-2-oxo-2H-chromen-3-yl)ethynyl)-[2,2'-bithiophen]-5-yl)acrylic acid (**9a**) were obtained.

^{1}H NMR (400 MHz, DMF-*d_7*) δ (ppm) 8.57 (s, 1H, H4/H9'), 8.38 (s, 1H, H4/H9'), 8.08 (d, *J* = 3.5 Hz, 1H, H5'/H6'/H7'/H8'), 7.73 (d, *J* = 3.7 Hz, 1H, H5'/H6'/H7'/H8'), 7.70 (d, *J* = 4.0 Hz, 1H, H5'/H6'/H7'/H8'), 7.53 (d, *J* = 4.5 Hz, 1H, H5'/H6'/H7'/H8'), 7.37 (s, 1H, H5/H8), 7.15 (s, 1H, H5/H8), 4.02 (s, 3H, H1'/H2'), 3.92 (s, 3H, H1'/H2').

HRMS-ESI(+) Calculated for $C_{25}H_{16}NO_6S_2$ $[M+H]^+$ 490.0414; Found 490.0408

2-cyano-3-(5’-((5,7-dimethoxy-2-oxo-*2H*-chromen-3-yl)ethynyl)-[2,2’-bithiophen]-5-yl)acrylic acid (**9b**):

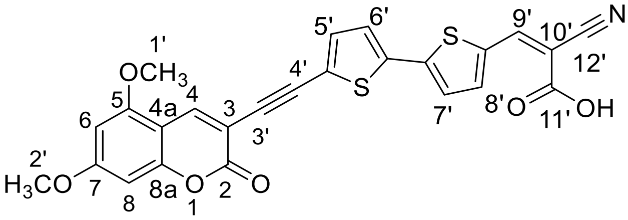

Starting from 23.2 mg (0.055 mmol, 1 eq.) of 5’-((5,7-dimethoxi-2-oxo-*2H*-chromen-3-yl)ethynyl)-[2,2’-bithiophene]-5-carbaldehyde (**5b**), 14.1 mg (52.5%) of 2-cyano-3-(5’-((5,7-dimethoxy-2-oxo-2H-chromen-3-yl)ethynyl)-[2,2’-bithiophen]-5-yl)acrylic acid (**9b**) were obtained.

^{1}H NMR (400 MHz, DMF-d_7) δ (ppm) 8.53 (s, 1H, H4/H9’), 8.27 (s, 1H, H4/H9’), 8.04 (s, 1H, H5’/H6’/H7’/H8’), 7.70 (br s, 1H, H5’/H6’/H7’/H8’), 7.67 (br s, 1H, H5’/H6’/H7’/H8’), 7.52 (br s, 1H, H5’/H6’/H7’/H8’), 6.65 (s, 1H, H6/H8), 6.61 (s, 1H, H6/H8), 4.04 (s, 3H, H1’/H2’), 3.99 (s, 3H, H1’/H2’).

HRMS-ESI(+) Calculated for $C_{25}H_{16}NO_6S_2$ $[M+H]^+$ 490.0414; Found 490.0406

2-cyano-3-(5-((6,7-dimethoxy-2-oxo-*2H*-chromen-3-yl)ethynyl)-thiophen-2-yl)acrylic acid (**10**):

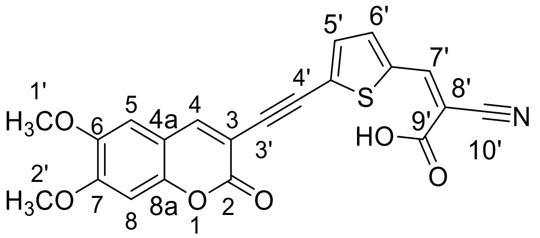

Starting from 28.1 mg (0.083 mmol, 1 eq.) of 5-((6,7-dimethoxy-2-oxo-*2H*-chromen-3-yl)ethynyl)-thiophene-2-carbaldehyde (**6**), 17.7 mg (50.8%) of 2-cyano-3-(5-((6,7-dimethoxy-2-oxo-*2H*-chromen-3-yl)ethynyl)-thiophen-2-yl)acrylic acid (**10**) were obtained.

^{1}H NMR (400 MHz, DMSO-d_6) δ (ppm) 8.50 (s, 1H, H4/H7’), 8.36 (s, 1H, H4/H7’), 7.98 (d, *J* = 3.8 Hz, 1H, H5’/H6’), 7.57 (d, *J* = 3.9 Hz, 1H, H5’/H6’), 7.22 (s, 1H, H5/H8), 7.11 (s, 1H, H5/H8), 3.89 (s, 3H, H1’/H2’), 3.81 (s, 3H, H1’/H2’).

HRMS-ESI(+) Calculated for $C_{21}H_{14}NO_6S$ $[M+H]^+$ 408.0536; Found 408.0529

2-cyano-3-(2-decyl-7-((6,7-dimethoxy-2-oxo-*2H*-chromen-3-yl)ethynyl)-*2H*-benzo[*d*][1,2,3]triazol-4-yl)acrylic acid (**11**):

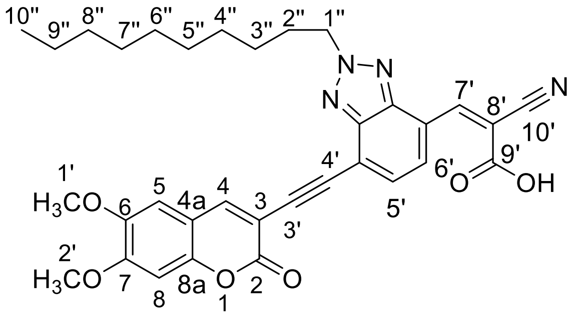

Starting from 51,7 mg (0.10 mmol, 1 eq.) of 2-decyl-7-((6,7-dimethoxy-2-oxo-2H-chromen-3-yl)ethynyl)-2H-benzo[d][1,2,3]triazole-4-carbaldehyde (**7**), 8.9 mg (50.8%) of 2-cyano-3-(2-decyl-7-((6,7-dimethoxy-2-oxo-*2H*-chromen-3-yl)ethynyl)-*2H*-benzo[*d*][1,2,3]triazol-4-yl)acrylic acid (**11**) were obtained.

^{1}H NMR (400 MHz, DMF-d_7) δ (ppm) 8.92 (s, 1H, H4/H7’), 8.55 (d, *J* = 8.0 Hz, 1H, H5’/H6’), 8.46 (s, 1H, H4/H7’), 7.97 (d, *J* = 7.8 Hz, 1H, H5’/H6’), 7.42 (s, 1H, H5/H8), 7.17 (s, 1H, H5/H8), 4.95 (t, *J* = 7.3 Hz, 2H, H1’’), 4.04 (s, 3H, H1’/H2’), 3.93 (s, 3H, H1’/H2’), 2.22 - 2.15 (m, 2H, H2’’), 1.44 - 1.14 (m, 20H, H3’-H9’’), 0.85 (t, *J* = 6.4 Hz, 5H, H10’’).

HRMS-ESI(+) Calculated for $C_{33}H_{35}N_4O_6$ $[M+H]^+$ 583.2551; Found 583.2542

4 Spectra

^{1}H-NMR (400 MHz, $CDCl_3$) spectrum of diethyl (2-oxo-2H-chromene-5,7-diyl) bis(carbonate)

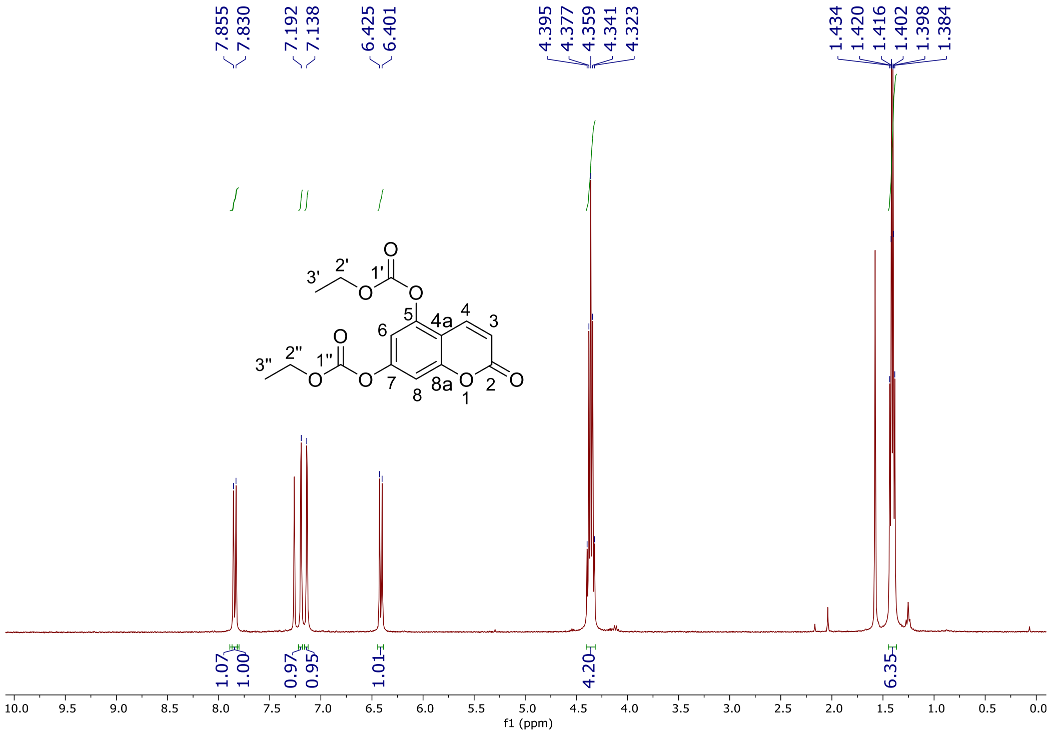

^{1}H-NMR (400 MHz, $CDCl_3$) spectrum of 3-bromo-5,7-dihydroxycoumarin

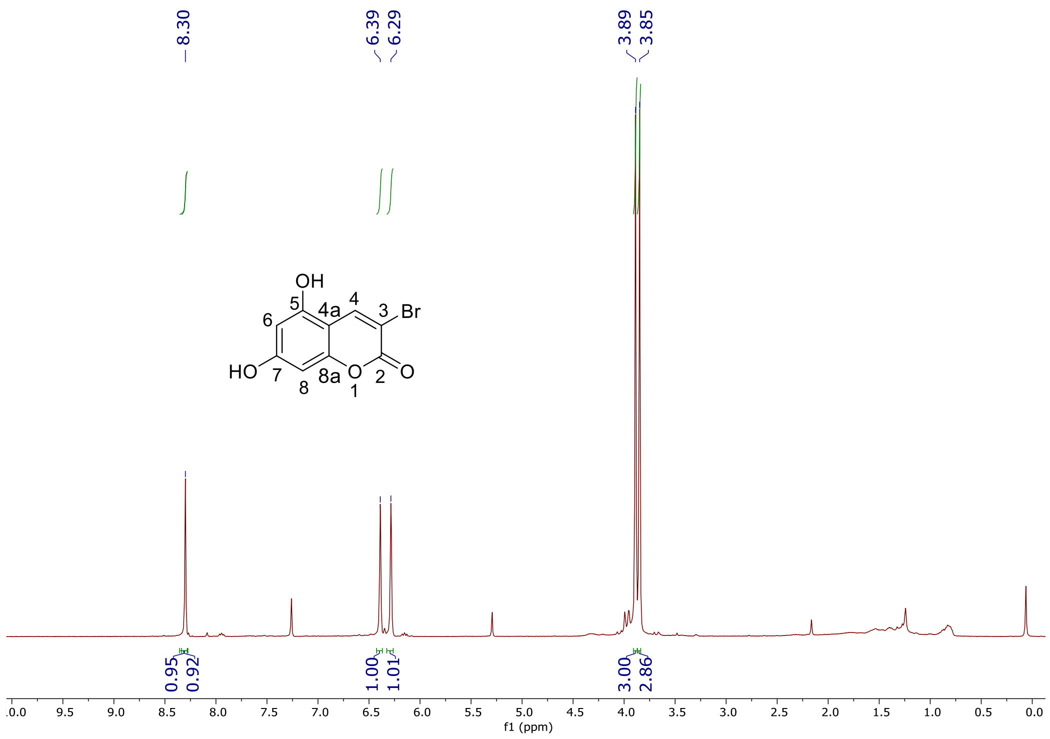

^{1}H-NMR (400 MHz, $CDCl_3$) spectrum of 3-bromo-6,7-dihydroxycoumarin (**1a**)

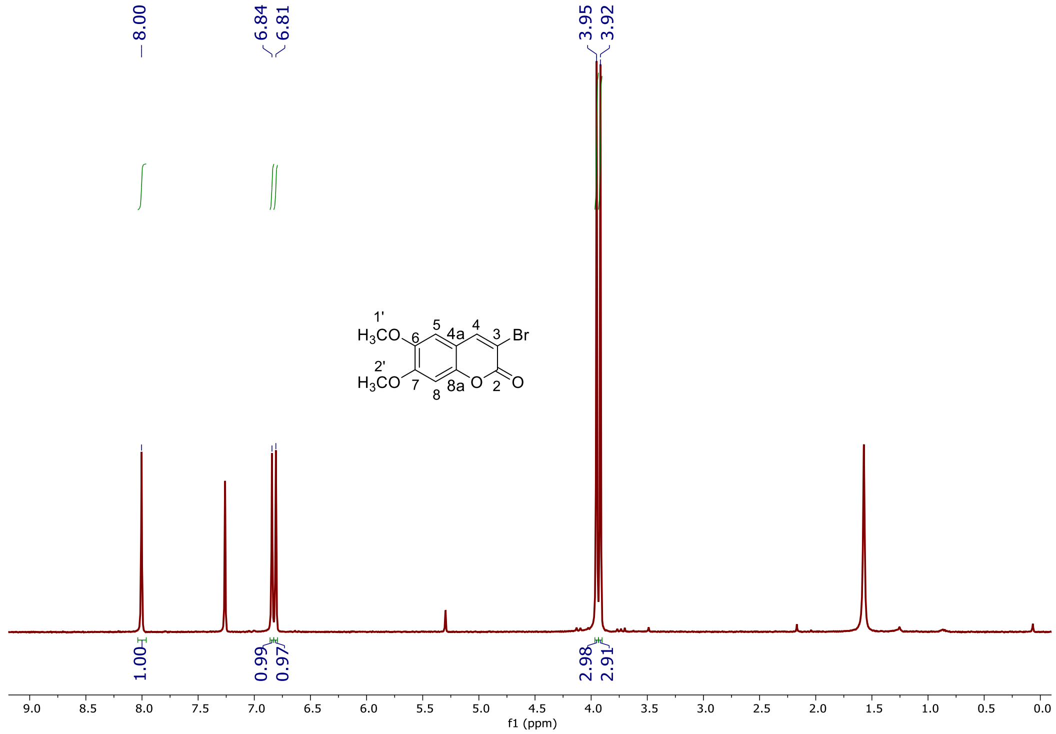

^{1}H-NMR (400 MHz, $CDCl_3$) spectrum of 6,7-dimethoxy-3-((trimethylsilyl)ethynyl)coumarin (**2a**)

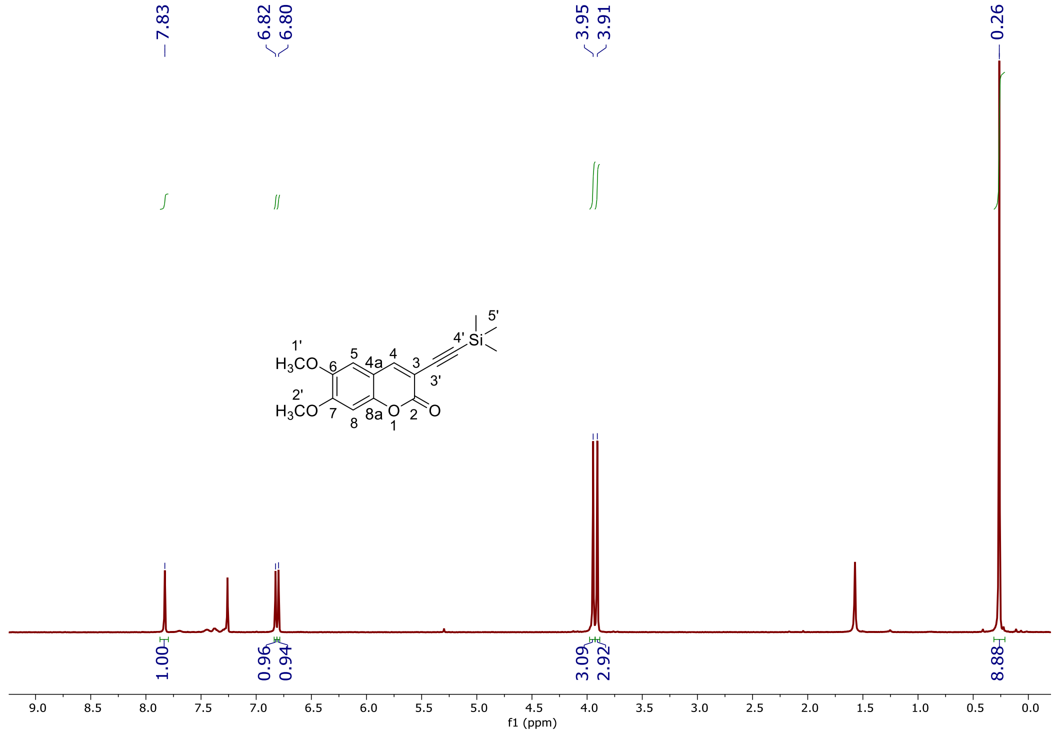

^{13}C-NMR (101 MHz, CDCl$_3$) spectrum of 6,7-dimethoxy-3-((trimethylsilyl)ethynyl)coumarin (**2a**)

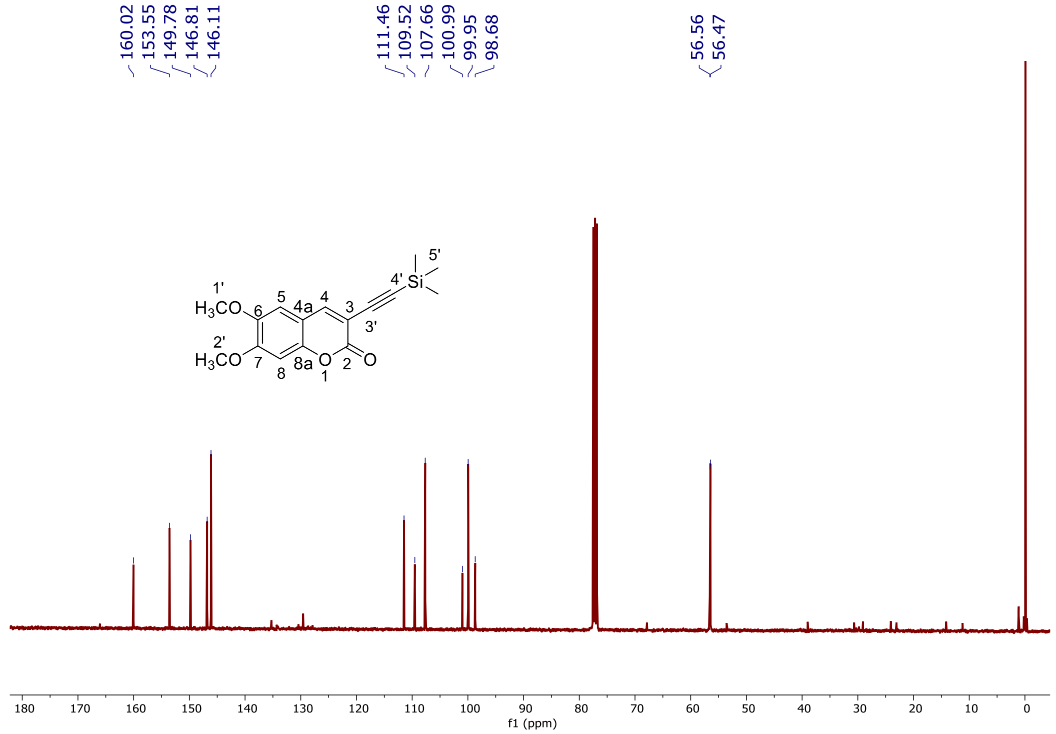

HRMS-ESI spectrum of 6,7-dimethoxy-3-((trimethylsilyl)ethynyl)coumarin (**2a**)

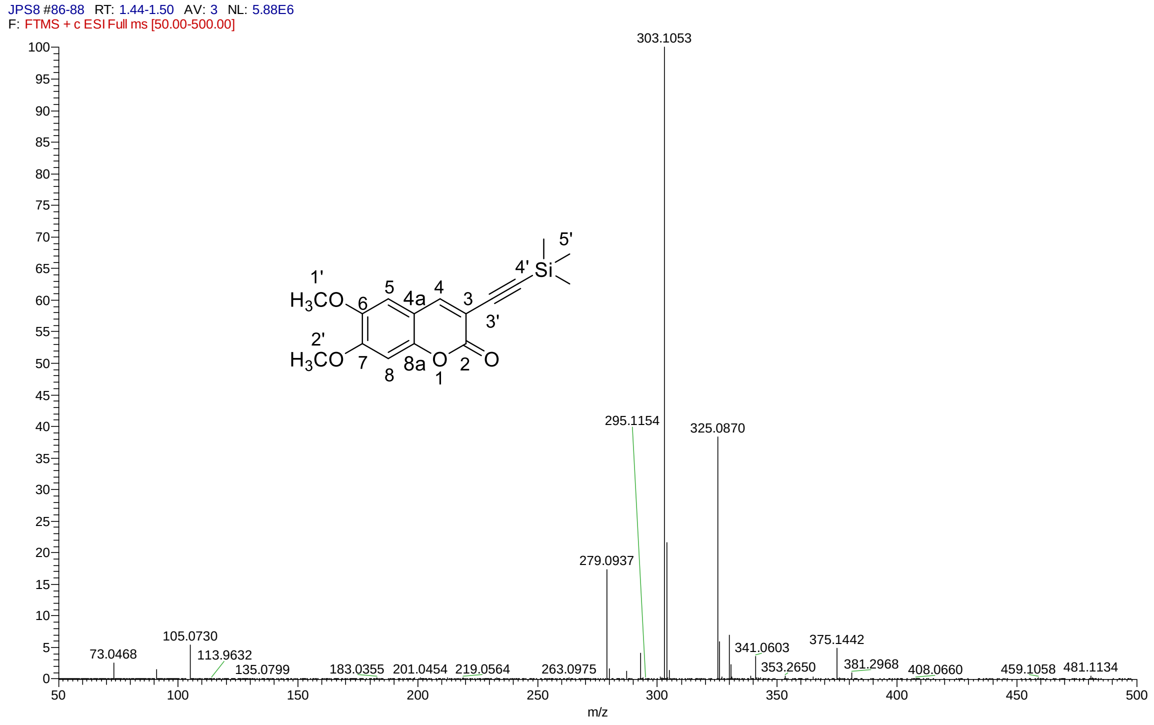

^{1}H-NMR (400 MHz, $CDCl_3$) spectrum of 5,7-dimethoxy-3-((trimethylsilyl)ethynyl)coumarin (**2b**)

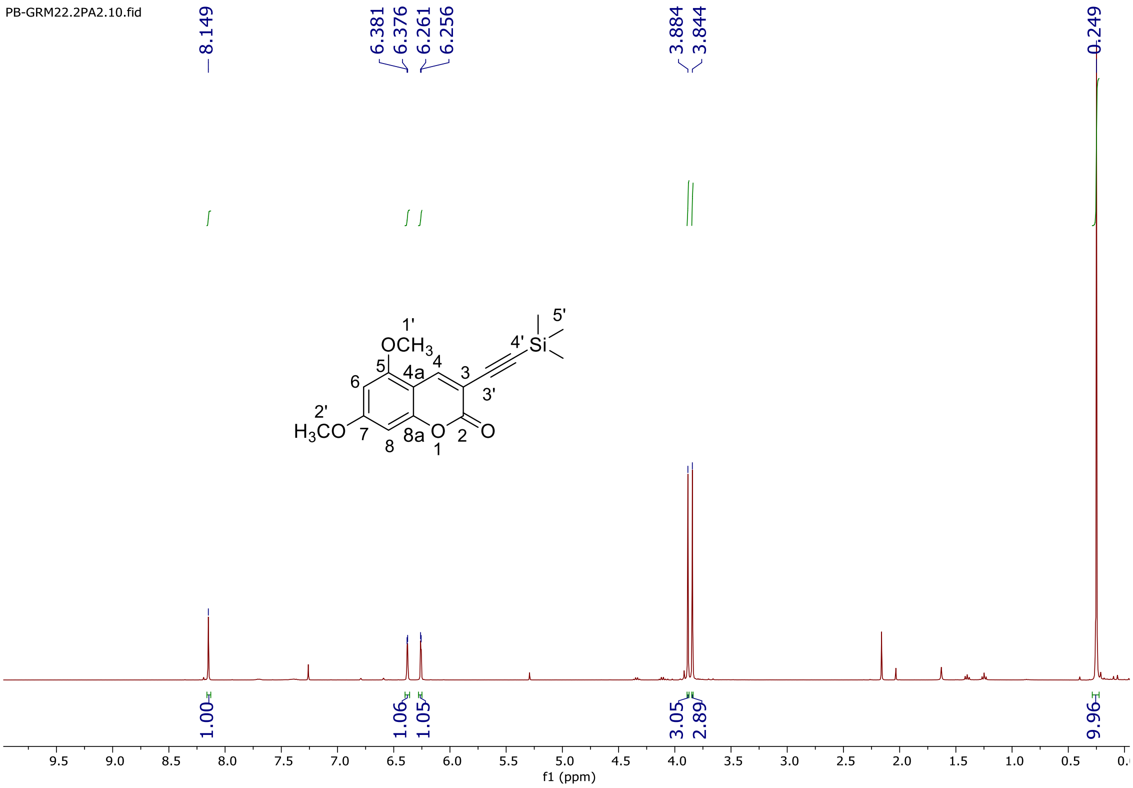

HRMS-ESI spectrum of 5,7-dimethoxy-3-((trimethylsilyl)ethynyl)coumarin (**2b**)

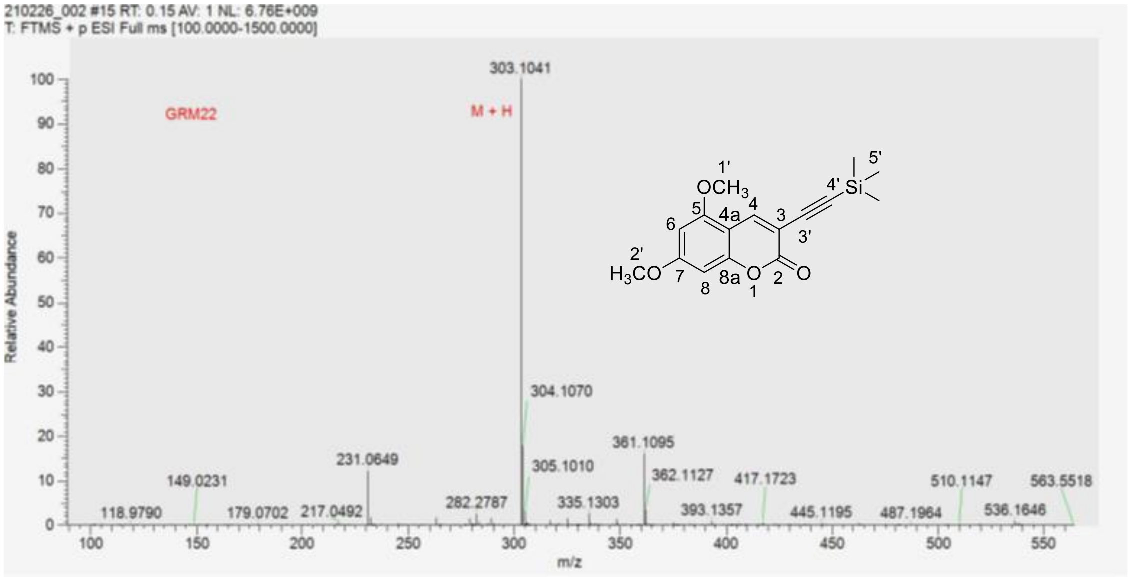

^{1}H-NMR (400 MHz, $CDCl_3$) spectrum of 5-bromothieno-[3,2-*b*]-tiophene-2-carbaldehyde

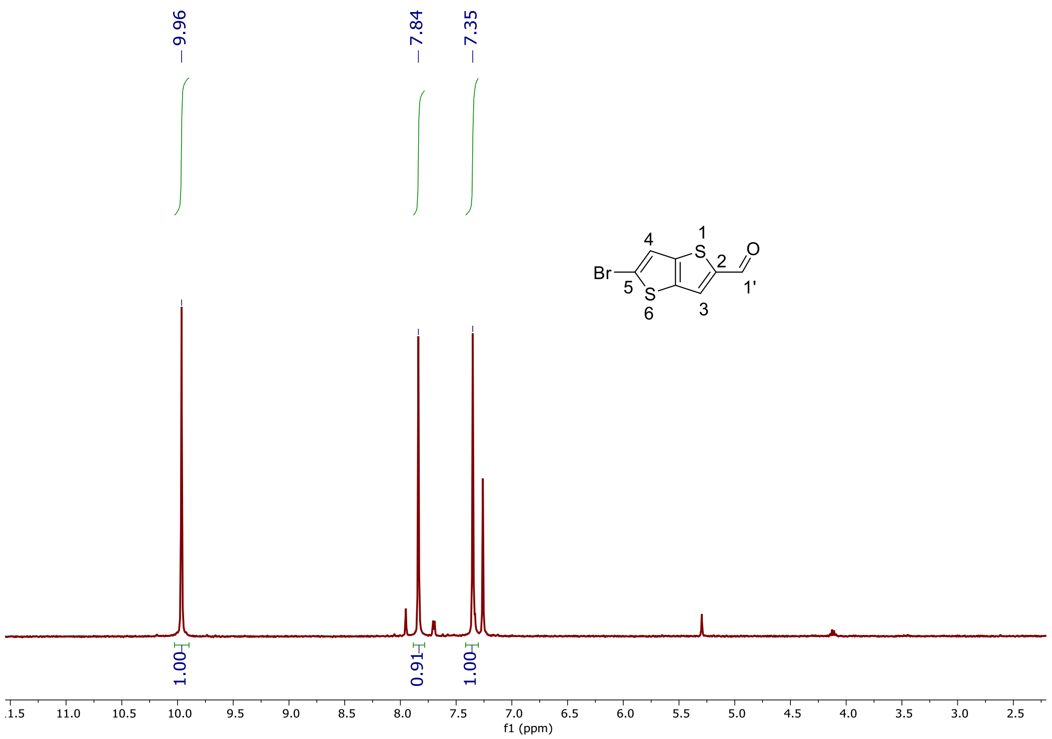

Mass spectra of 5-bromothieno-[3,2-*b*]-tiophene-2-carbaldehyde

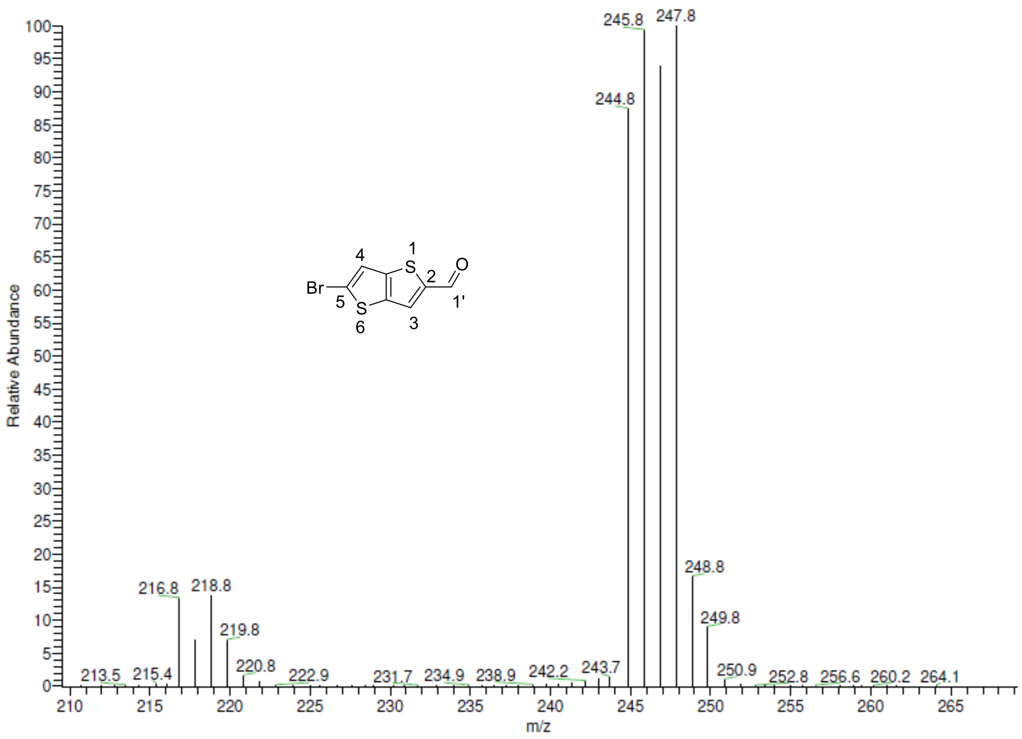

^{1}H-NMR (400 MHz, $CDCl_3$) spectrum of 2-decyl-*2H*-benzo[*d*][1,2,3]triazole

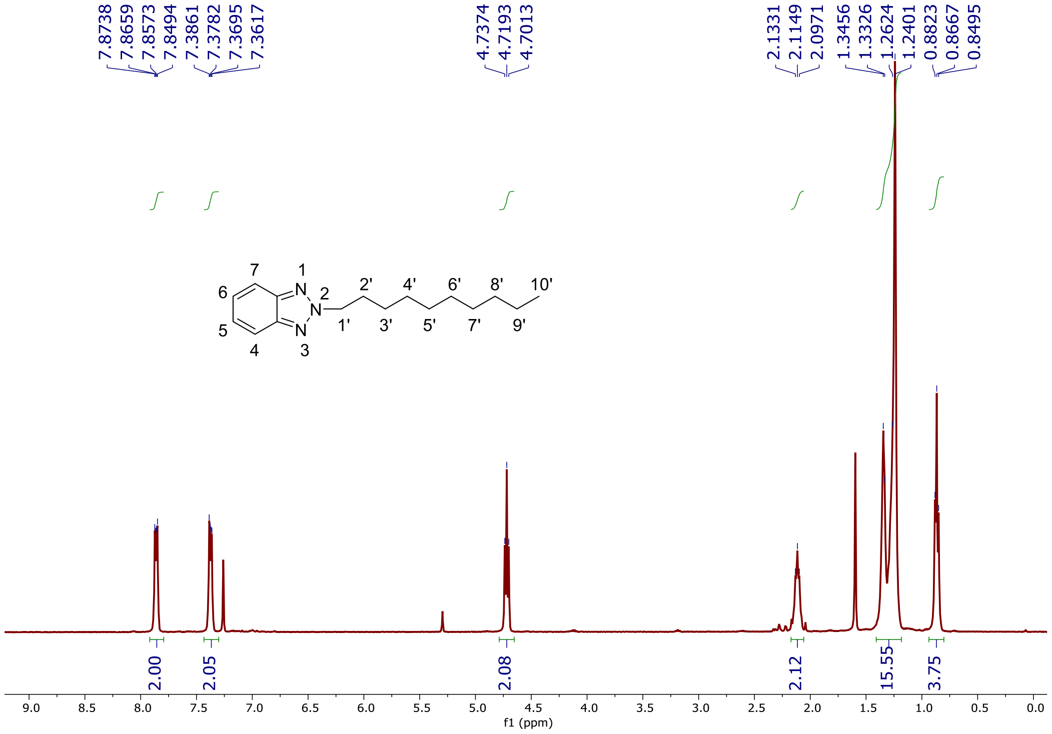

^{1}H-NMR (400 MHz, $CDCl_3$) spectrum of 4,7-dibromo-2-decyl-*2H*-benzo[*d*][1,2,3]triazole

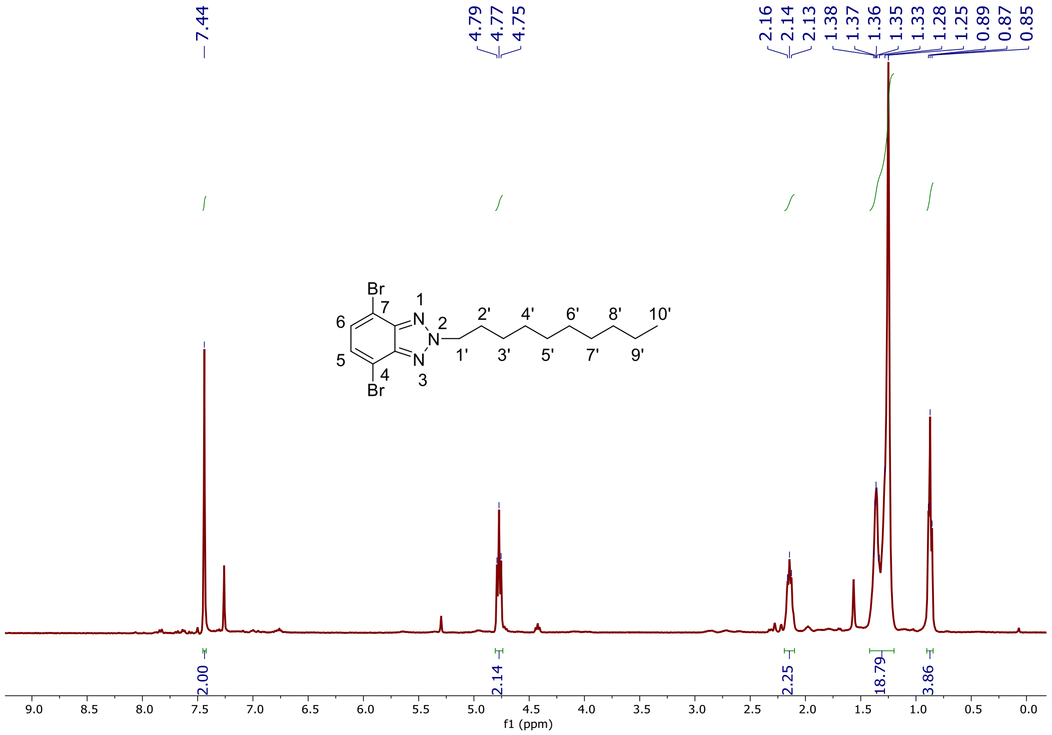

^{1}H-NMR (400 MHz, $CDCl_3$) spectrum of 7-dibromo-2-decyl-2H-benzo[d][1,2,3]triazole-4-carbaldehyde

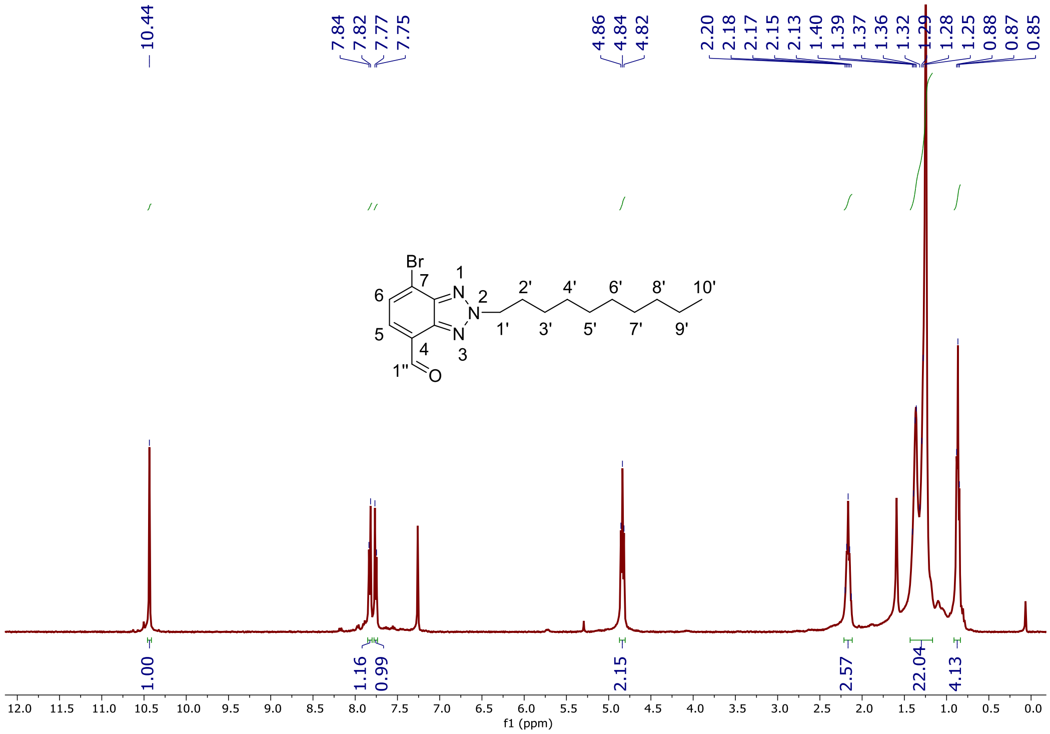

^{1}H-NMR (400 MHz, $CDCl_3$) spectrum of 5-((6,7-dimethoxy-2-oxo-2H-chromen-3-yl)ethynyl)thieno[3,2-b]tiophene-2-carbaldehyde (**4**)

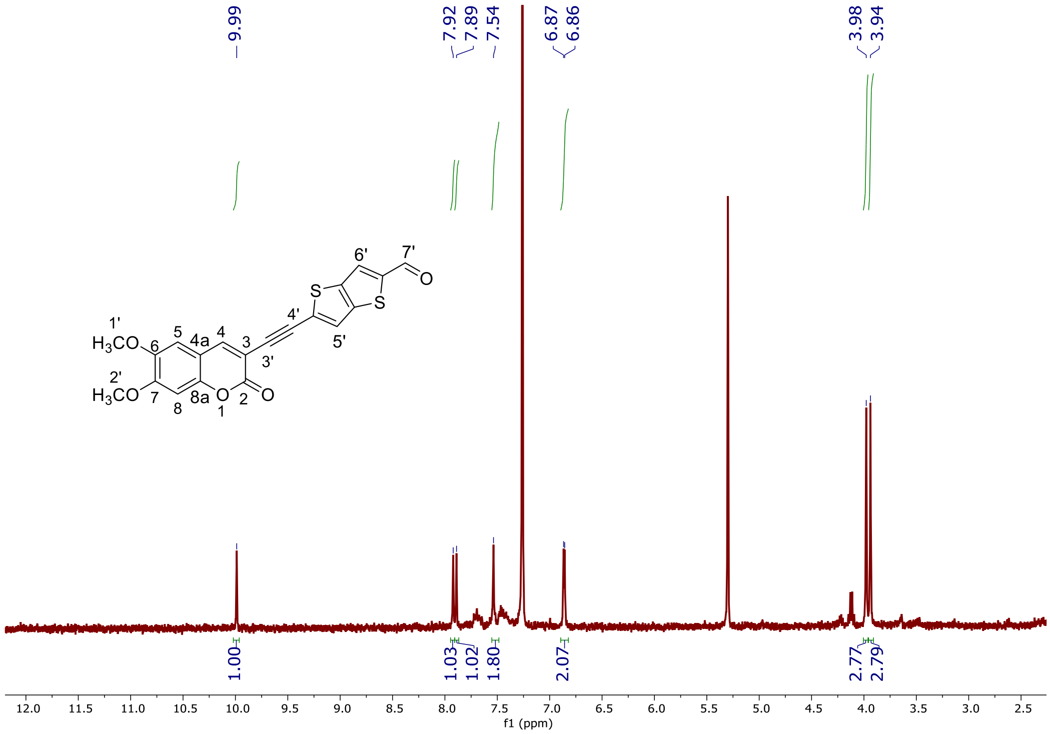

HRMS-ESI spectrum of 5-((6,7-dimethoxy-2-oxo-2H-chromen-3-yl)ethynyl)thieno[3,2-b]tiophene-2-carbaldehyde (**4**)

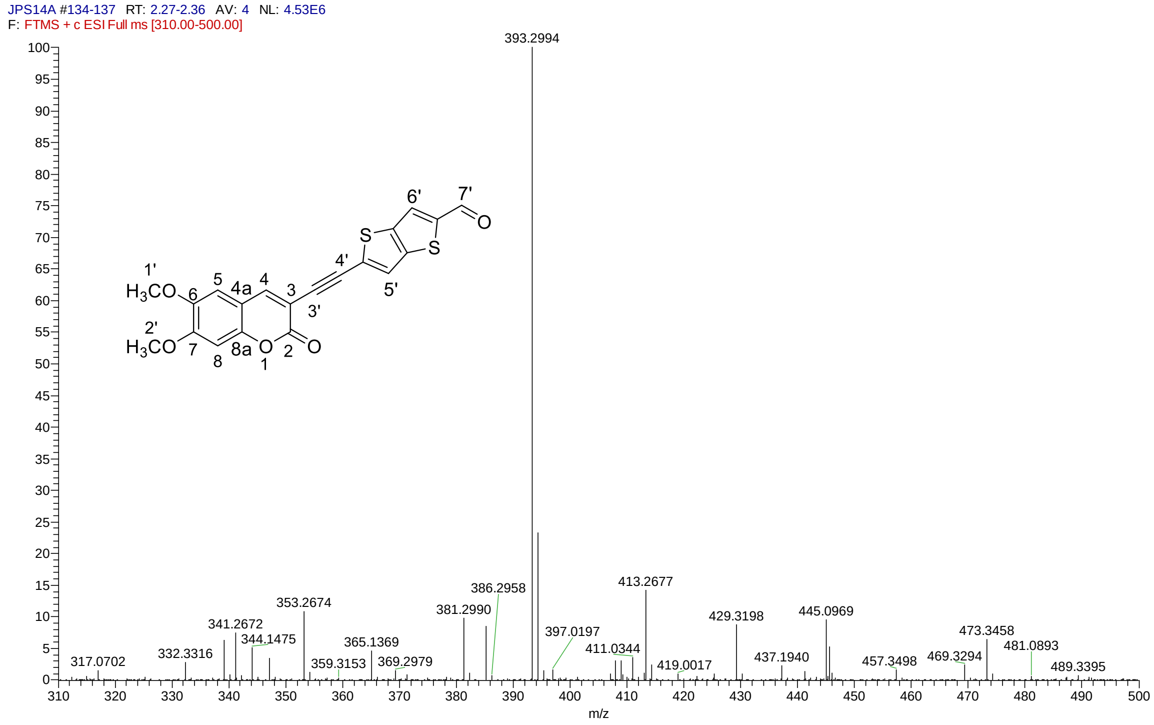

^{1}H-NMR (400 MHz, CD_2Cl_2) spectrum of 5'-((6,7-dimethoxi-2-oxo-2H-chromen-3-yl)ethynyl)-[2,2'-bithiophene]-5-carbaldehyde (**5a**)

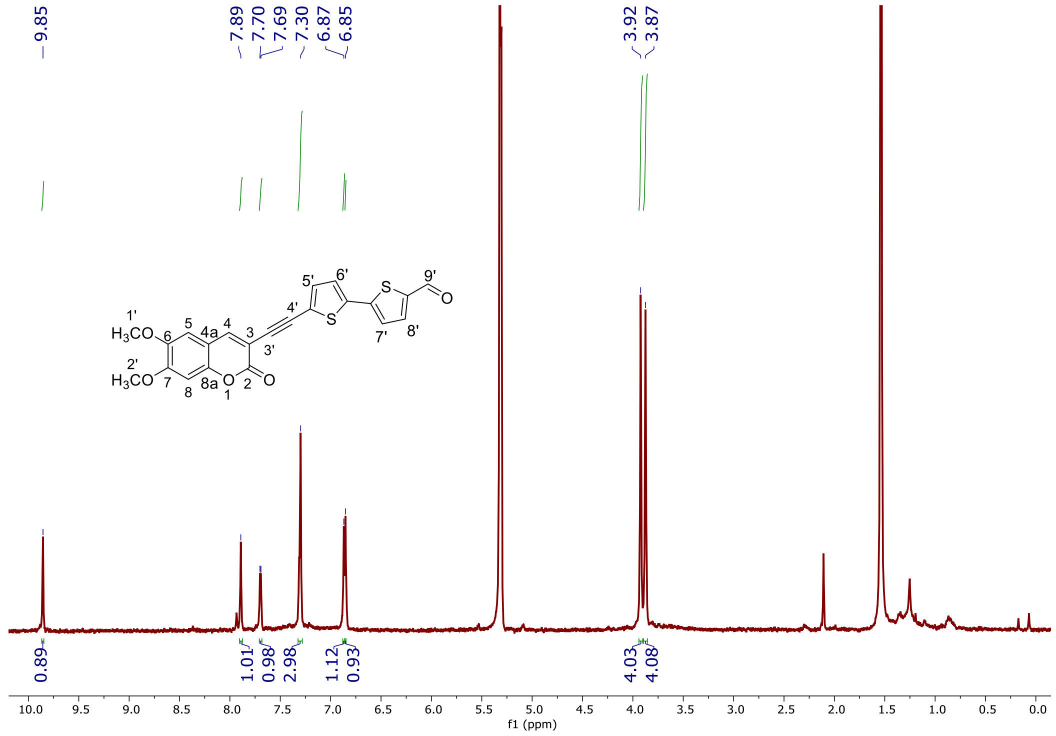

HRMS-ESI spectrum of 5’-((6,7-dimethoxi-2-oxo-2H-chromen-3-yl)ethynyl)-[2,2’-bithiophene]-5-carbaldehyde (**5a**)

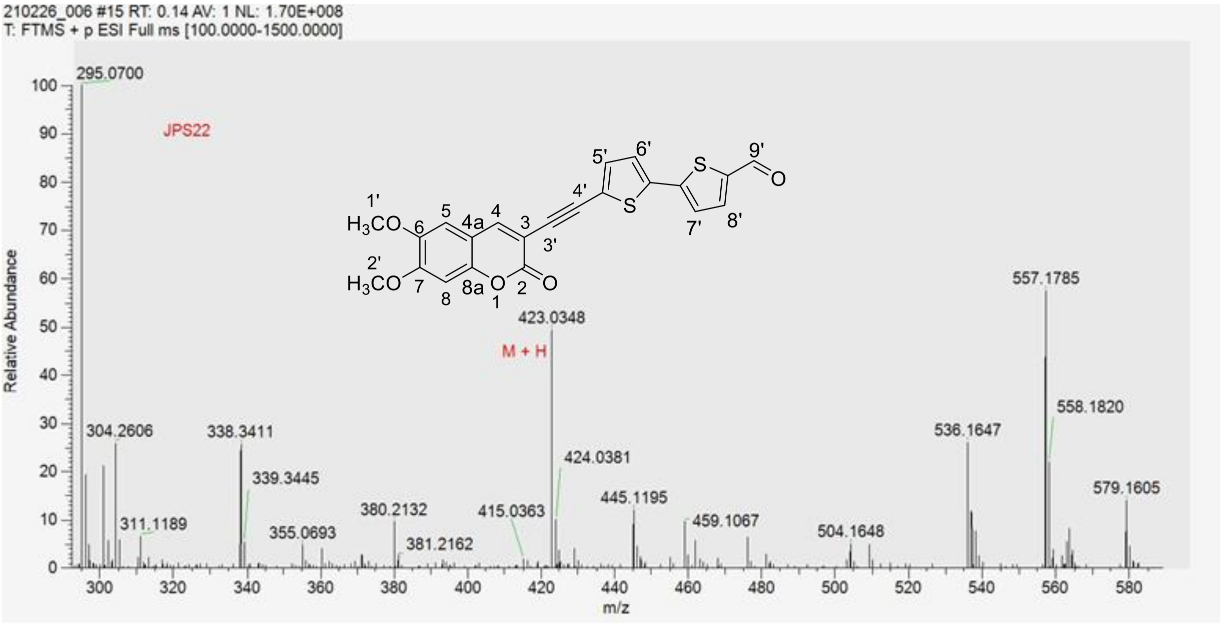

^{1}H-NMR (400 MHz, DMF-d_7) spectrum of 5’-((5,7-dimethoxi-2-oxo-2H-chromen-3-yl)ethynyl)-[2,2’-bithiophene]-5-carbaldehyde (**5b**)

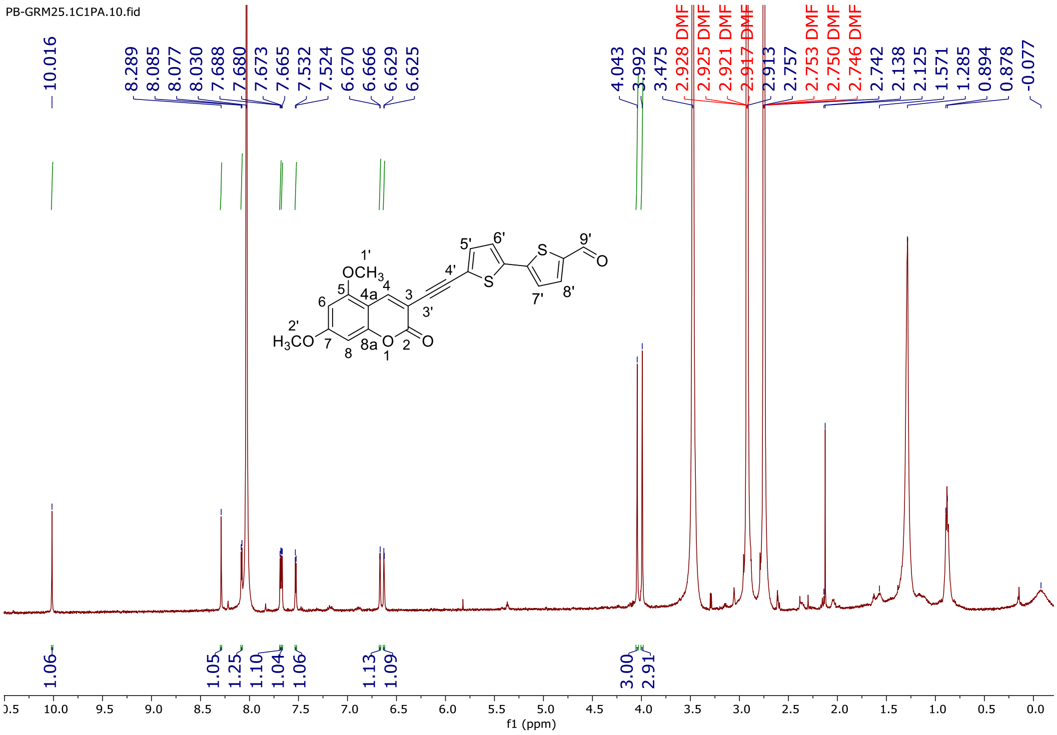

HRMS-ESI spectrum of 5'-((5,7-dimethoxi-2-oxo-2H-chromen-3-yl)ethynyl)-[2,2'-bithiophene]-5-carbaldehyde (**5b**)

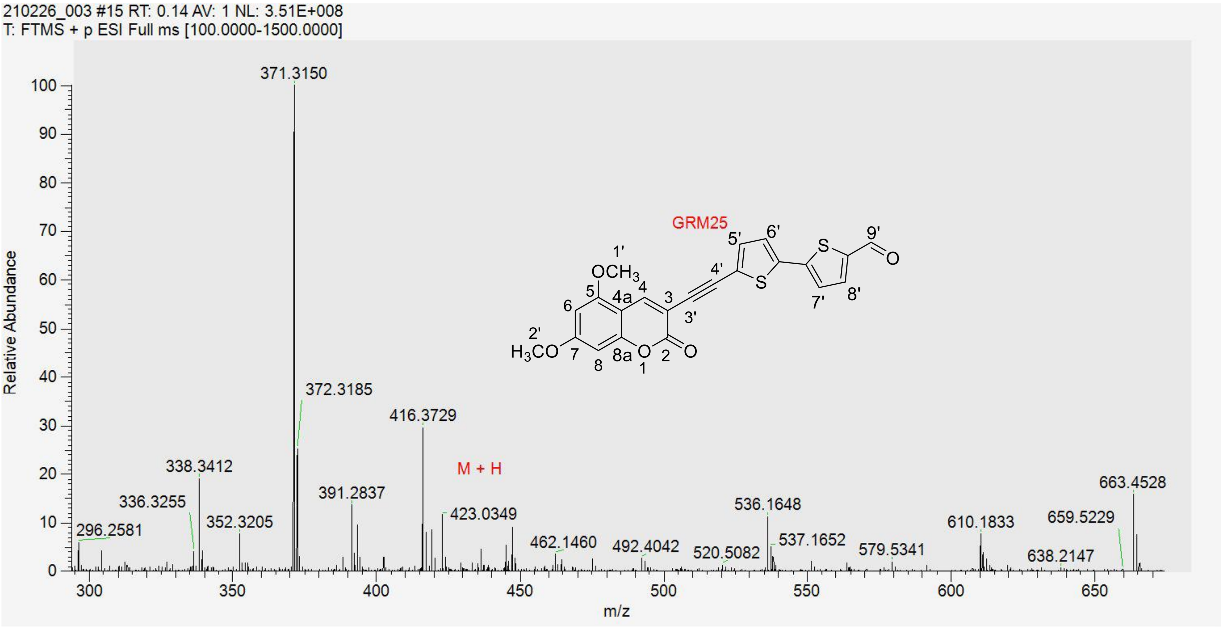

^{1}H-NMR (400 MHz, CD_2Cl_2) spectrum of 5-((6,7-dimethoxi-2-oxo-2H-chromen-3-yl)ethynyl)-thiophene-5-carbaldehyde (**6**)

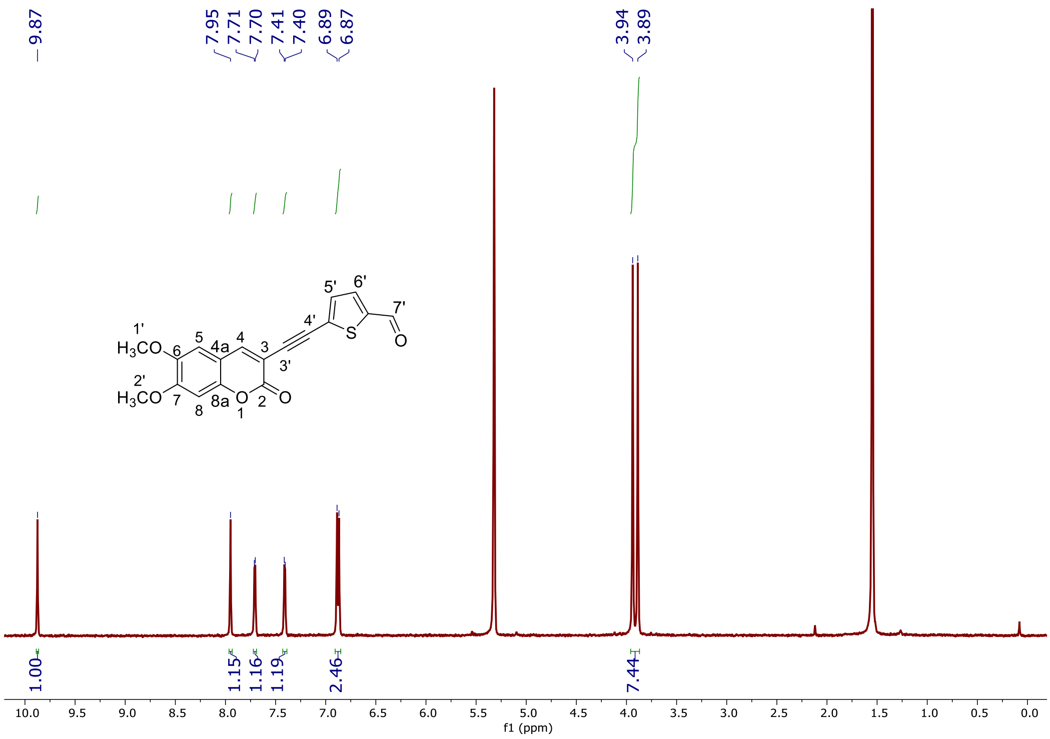

HRMS-ESI spectrum of 5-((6,7-dimethoxi-2-oxo-2H-chromen-3-yl)ethynyl)-thiophene-5-carbaldehyde (**6**)

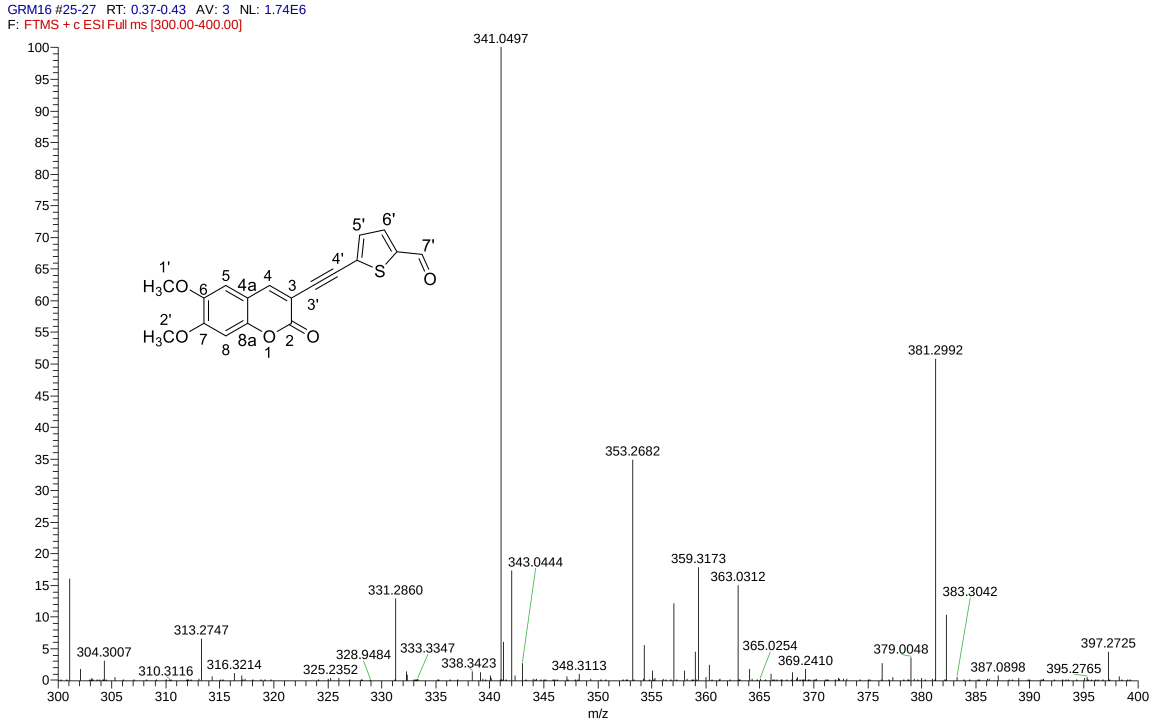

^{1}H-NMR (400 MHz, CD_2Cl_2) spectrum of 2-decyl-7-((6,7-dimethoxy-2-oxo-2H-chromen-3-yl)ethynyl)-2H-benzo[d][1,2,3]triazole-4-carbaldehyde (**7**)

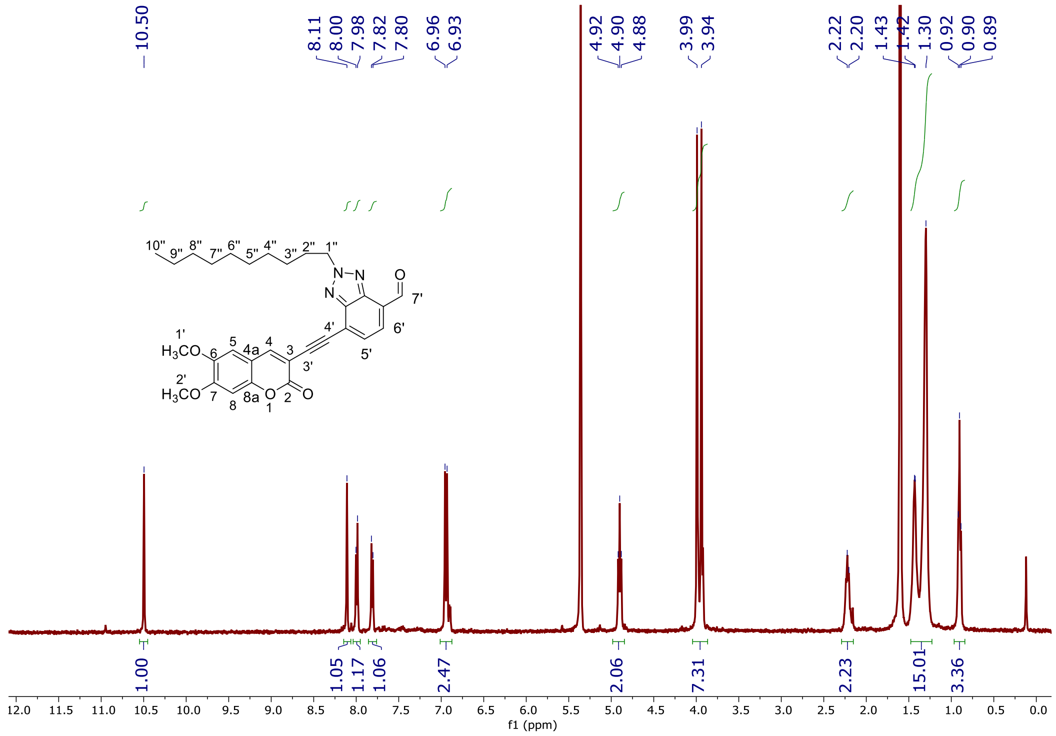

HRMS-ESI spectrum of 2-decyl-7-((6,7-dimethoxy-2-oxo-2H-chromen-3-yl)ethynyl)-2H-benzo[d][1,2,3]triazole-4-carbaldehyde (**7**)

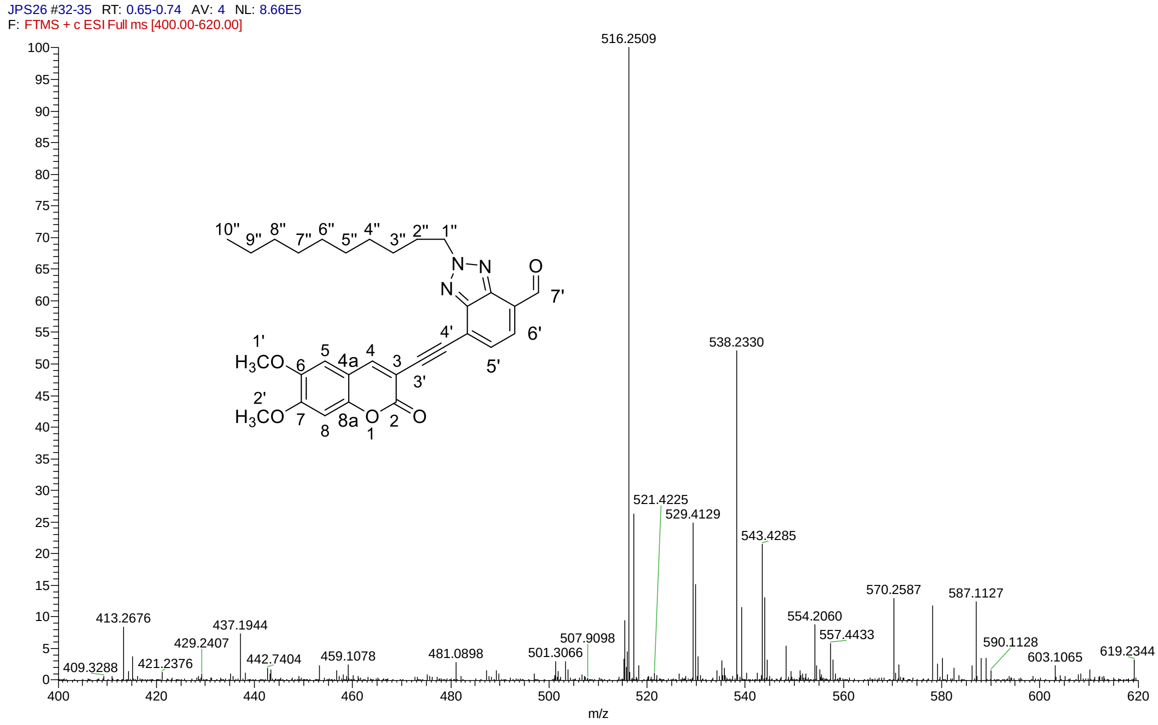

^{1}H-NMR (400 MHz, DMSO-d_6) spectrum of 2-cyano-3-(5-((6,7-dimethoxy-2-oxo-2H-chromen-3-yl)ethynyl)thieno[3,2-b]thiophen-2-yl)acrylic acid (**8**)

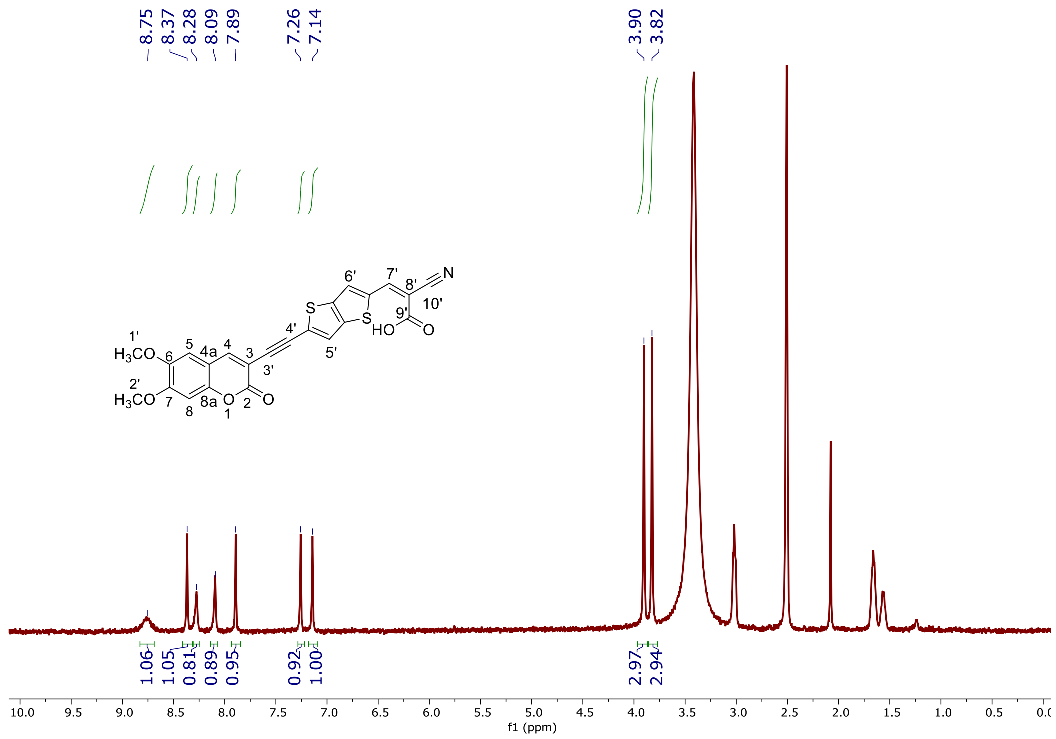

HRMS-ESI spectrum of 2-cyano-3-(5-((6,7-dimethoxy-2-oxo-2H-chromen-3-yl)ethynyl)thieno[3,2-b]thiophen-2-yl)acrylic acid (**8**)

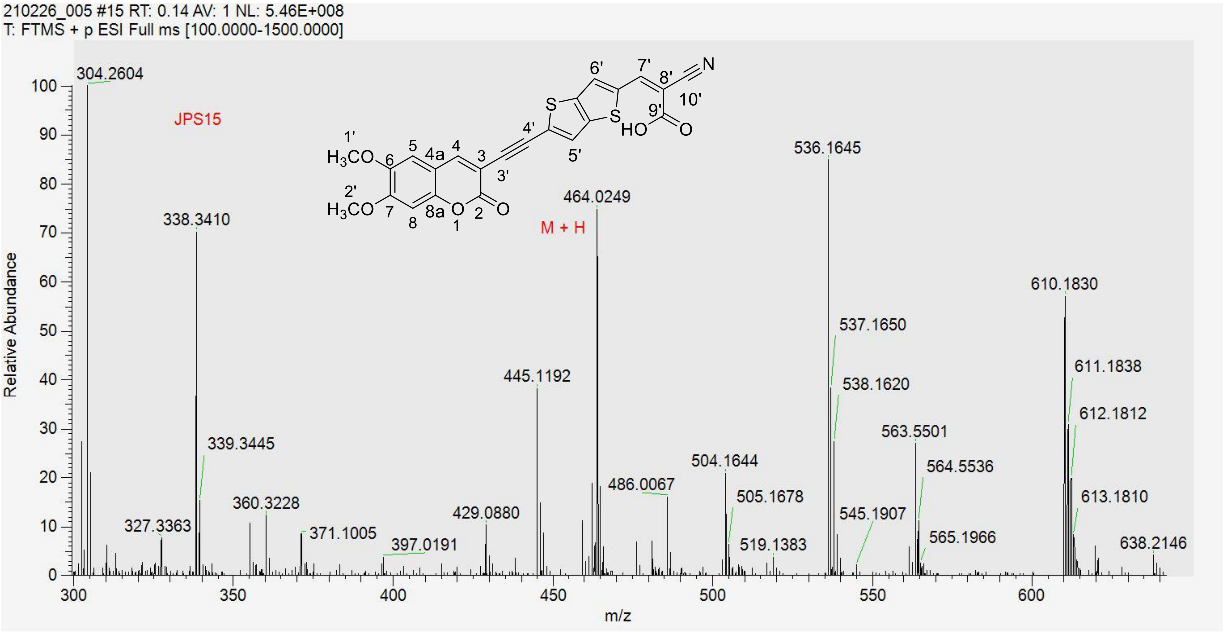

^{1}H-NMR (400 MHz, DMF-d_7) spectrum of 2-cyano-3-(5′-((6,7-dimethoxy-2-oxo-2H-chromen-3-yl)ethynyl)-[2,2′-bithiophen]-5-yl)acrylic acid (**9a**)

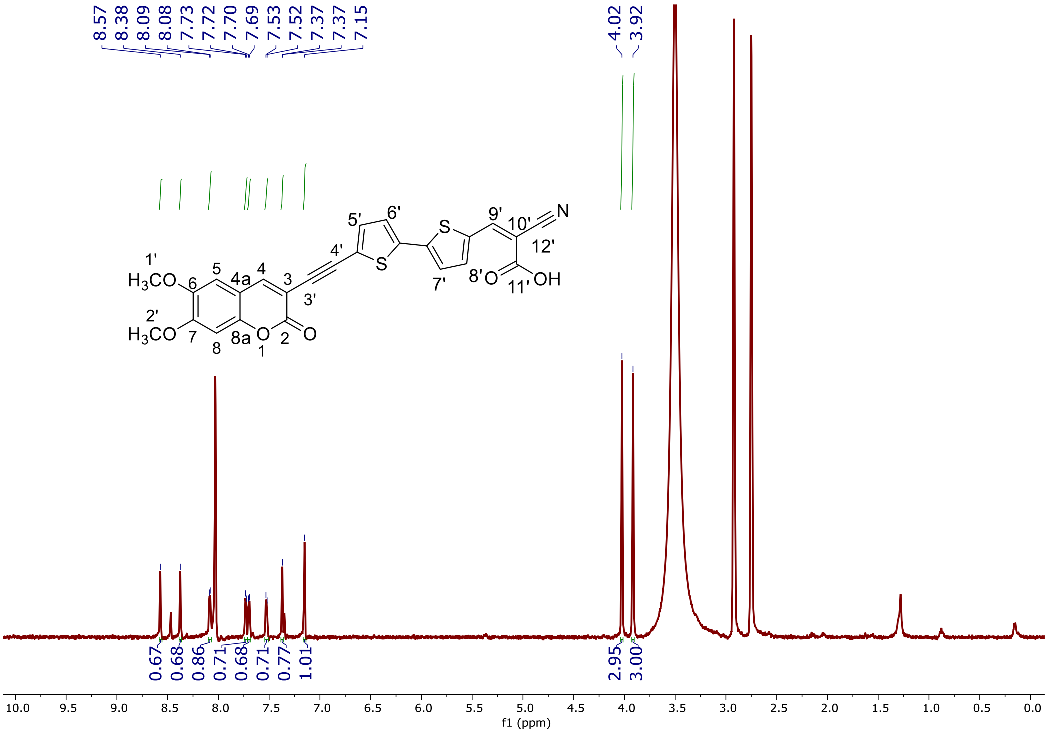

HRMS-ESI spectrum of 2-cyano-3-(5’-((6,7-dimethoxy-2-oxo-2H-chromen-3-yl)ethynyl)-[2,2’-bithiophen]-5-yl)acrylic acid (**9a**)

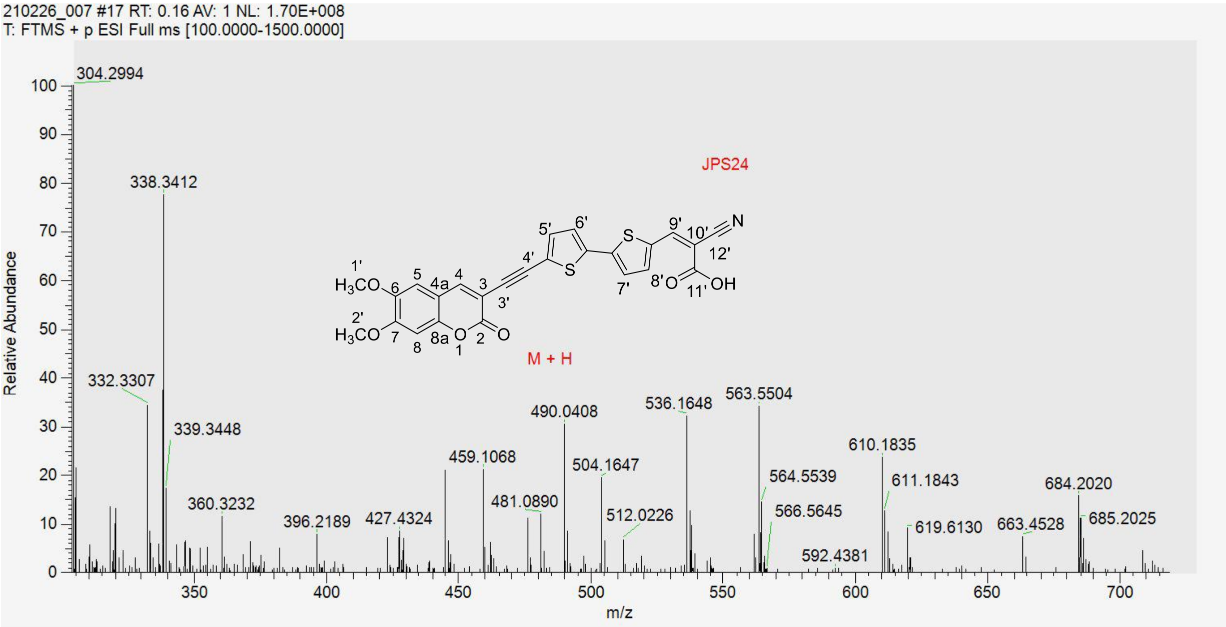

^{1}H-NMR (400 MHz, DMF-d_7) spectrum of 2-cyano-3-(5’-((5,7-dimethoxy-2-oxo-2H-chromen-3-yl)ethynyl)-[2,2’-bithiophen]-5-yl)acrylic acid (**9b**)

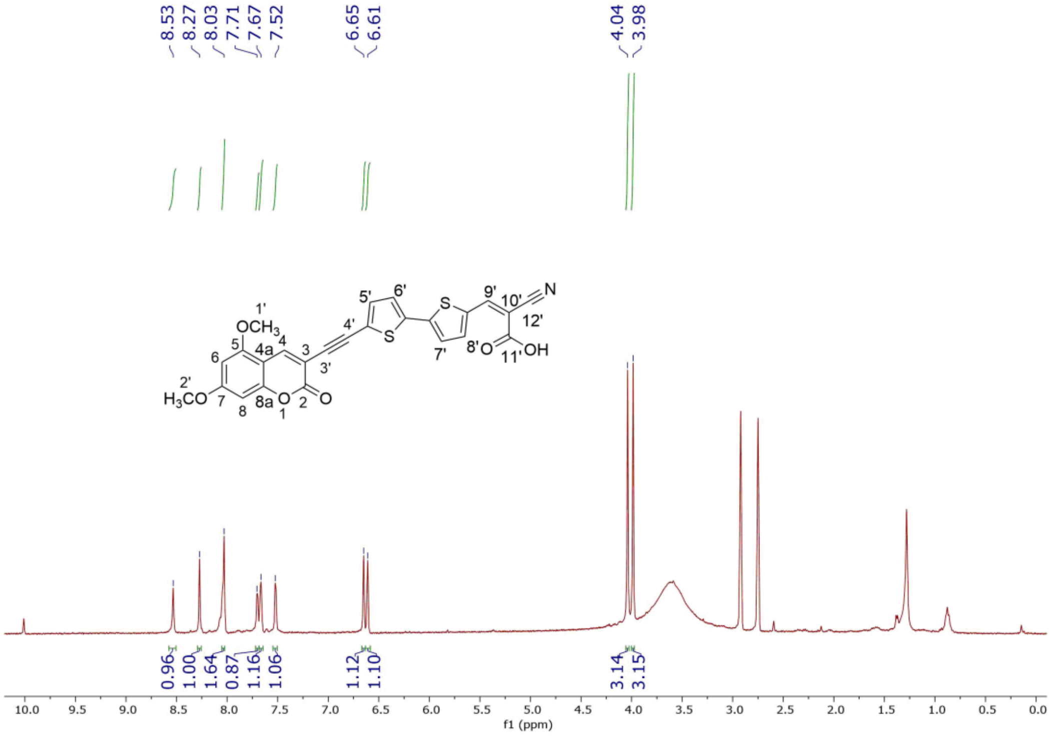

HRMS-ESI spectrum of 2-cyano-3-(5’-((5,7-dimethoxy-2-oxo-2H-chromen-3-yl)ethynyl)-[2,2’-bithiophen]-5-yl)acrylic acid (**9b**)

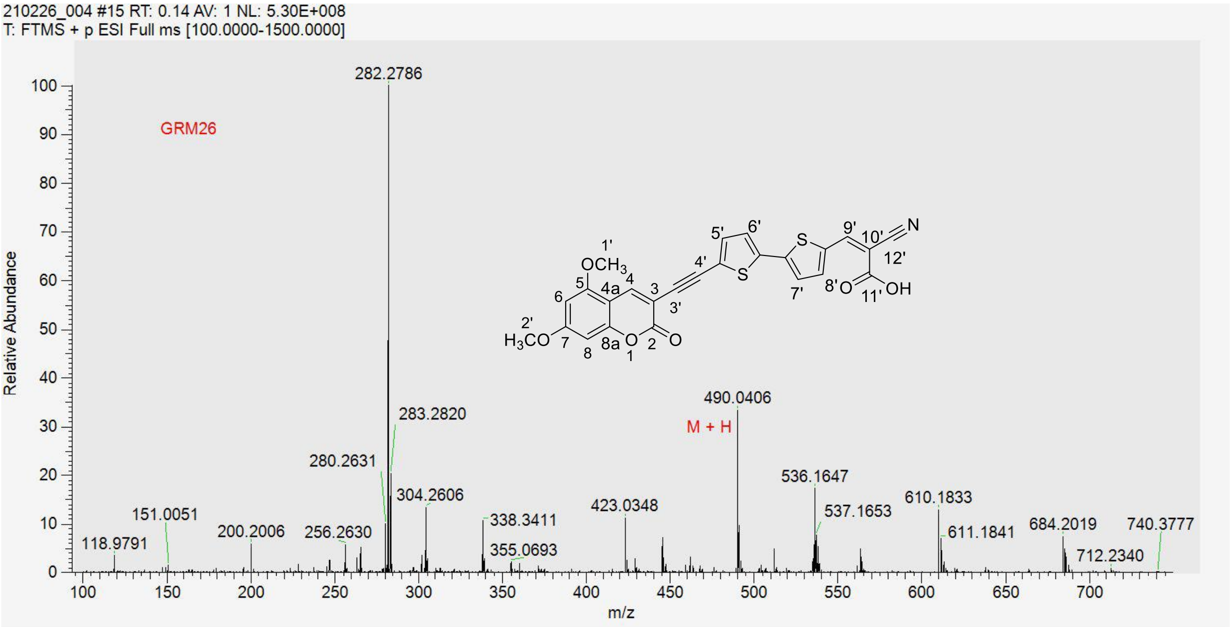

^{1}H-NMR (400 MHz, DMSO-d_6) spectrum of 2-cyano-3-(5-((6,7-dimethoxy-2-oxo-2H-chromen-3-yl)ethynyl)-thiophen-2-yl)acrylic acid (**10**)

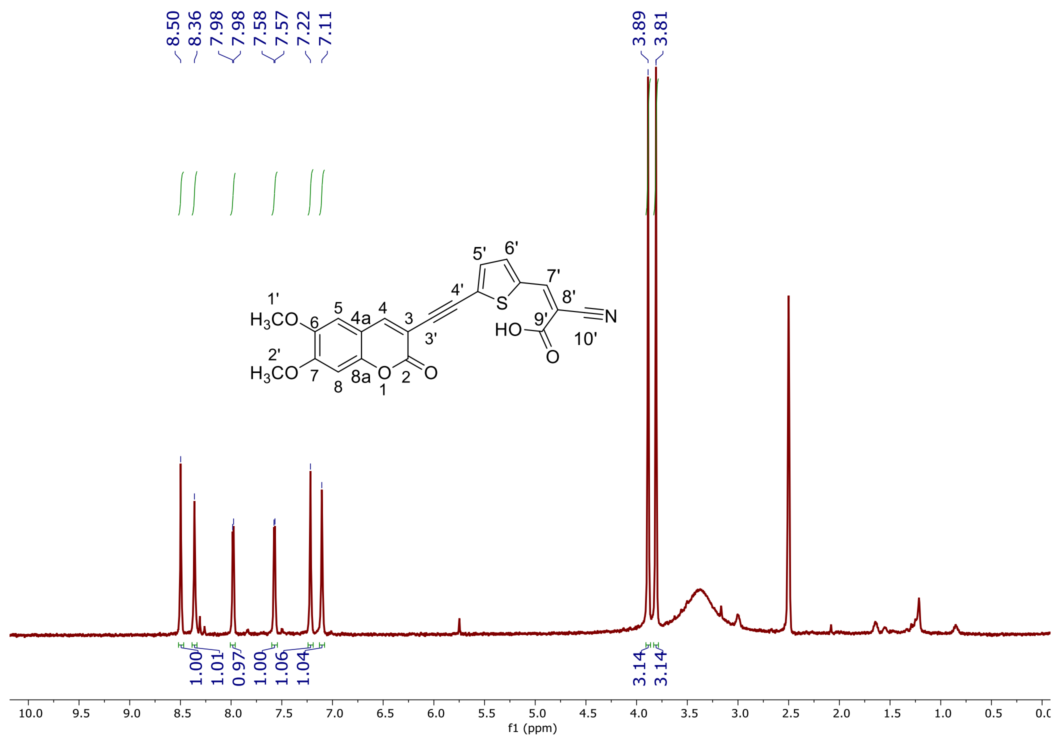

HRMS-ESI spectrum of 2-cyano-3-(5-((6,7-dimethoxy-2-oxo-2H-chromen-3-yl)ethynyl)-thiophen-2-yl)acrylic acid (**10**)

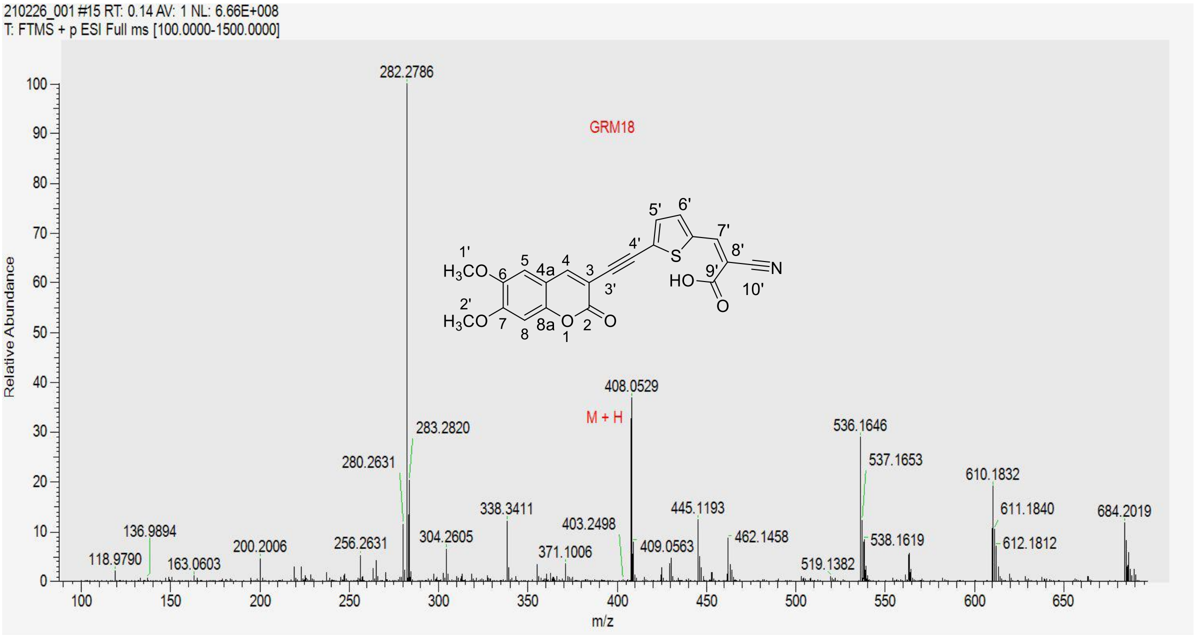

^{1}H-NMR (400 MHz, DMF-d_7) spectrum of 2-cyano-3-(2-decyl-7-((6,7-dimethoxy-2-oxo-2H-chromen-3-yl)ethynyl)-2H-benzo[d][1,2,3]triazol-4-yl)acrylic acid (**11**)

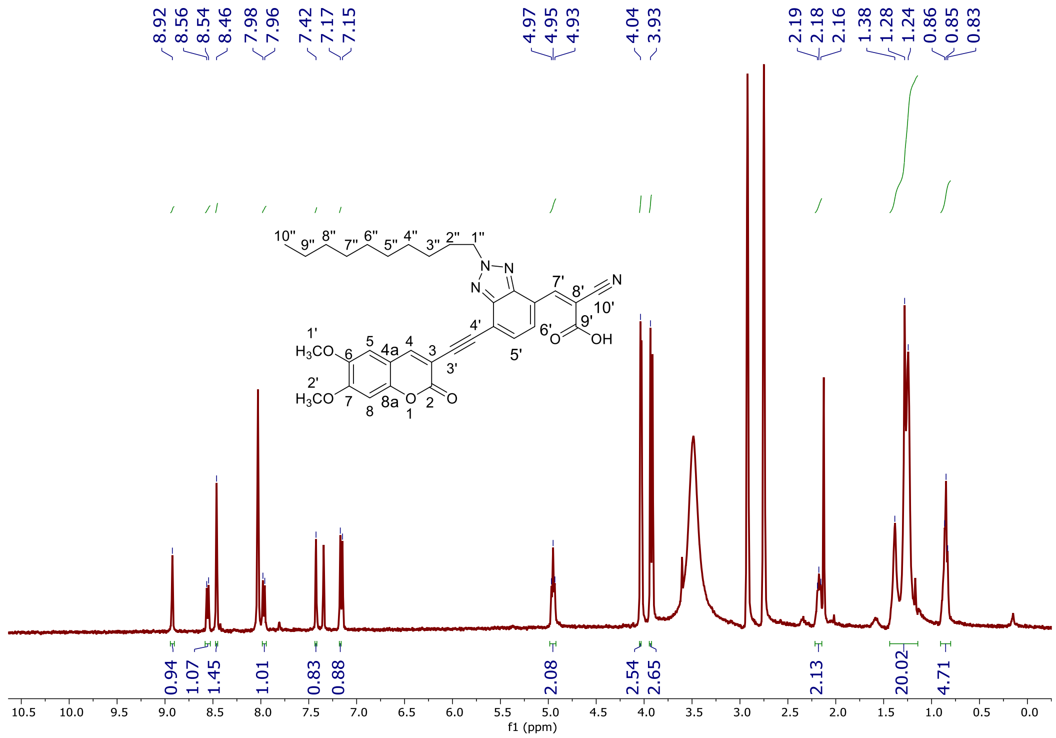

HRMS-ESI spectrum of 2-cyano-3-(2-decyl-7-((6,7-dimethoxy-2-oxo-2H-chromen-3-yl)ethynyl)-2H-benzo[d][1,2,3]triazol-4-yl)acrylic acid (**11**)

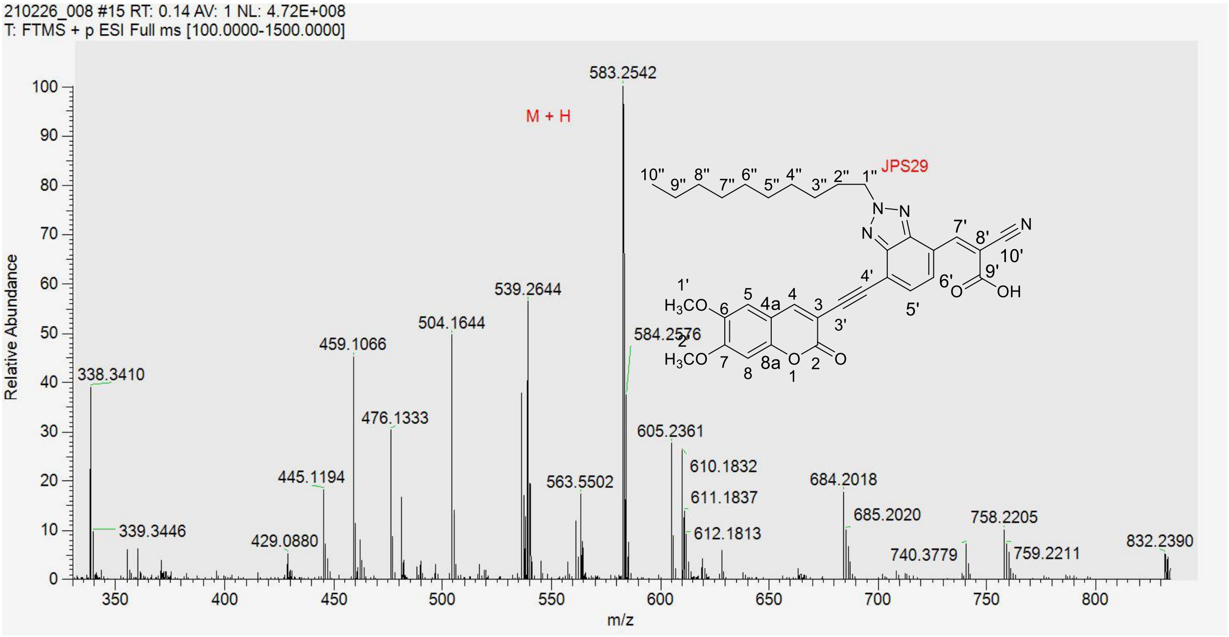

Supplement: Supplementary file 1 [file molecules-26-02934-s001.zip › molecules-1186206-supplementary.pdf]
